# *comCDE* (Competence) Operon Is Regulated by CcpA in Streptococcus pneumoniae D39

**DOI:** 10.1128/spectrum.00012-23

**Published:** 2023-04-10

**Authors:** Yapeng Zhang, Jinghui Zhang, Jiangming Xiao, Hanyi Wang, Rui Yang, Xinlin Guo, Yuqiang Zheng, Yibing Yin, Xuemei Zhang

**Affiliations:** a Key Laboratory of Diagnostic Medicine Designated by the Ministry of Education, Department of Laboratory Medicine, Chongqing Medical University, Chongqing, China; b Zybio Inc., Chongqing, China; c Department of Medicine Laboratory, Children’s Hospital of Chongqing Medical University, Chongqing, China; Department of Clinical Laboratory, Peking University People’s Hospital, Beijing, China

**Keywords:** *Streptococcus pneumoniae* D39, competence, transformation, CcpA

## Abstract

Natural transformation plays an important role in the formation of drug-resistant bacteria. Exploring the regulatory mechanism of natural transformation can aid the discovery of new antibacterial targets and reduce the emergence of drug-resistant bacteria. Competence is a prerequisite of natural transformation in Streptococcus pneumoniae, in which *comCDE* operon is the core regulator of competence. To date, only ComE has been shown to directly regulate *comCDE* transcription. In this study, a transcriptional regulator, the catabolite control protein A (CcpA), was identified that directly regulated *comCDE* transcription. We confirmed that CcpA binds to the *cis*-acting catabolite response elements (cre) in the *comCDE* promoter region to regulate *comCDE* transcription and transformation. Moreover, CcpA can coregulate *comCDE* transcription with phosphorylated and dephosphorylated ComE. Regulation of *comCDE* transcription and transformation by CcpA was also affected by carbon source signals. Together, these insights demonstrate the versatility of CcpA and provide a theoretical basis for reducing the emergence of drug-resistant bacteria.

**IMPORTANCE** Streptococcus pneumoniae is a major cause of bacterial infections in humans, such as pneumonia, bacteremia, meningitis, otitis media, and sinusitis. Like most streptococci, S. pneumoniae is naturally competent and employs this ability to augment its adaptive evolution. The current study illustrates CcpA, a carbon catabolite regulator, can participate in the competence process by regulating *comCDE* transcription, and this process is regulated by different carbon source signals. These hidden abilities are likely critical for adaptation and colonization in the environment.

## INTRODUCTION

Natural transformation refers to the process in which bacteria become naturally competent to actively absorb exposed DNA fragments from the environment and integrate them into their own genomes (1). This process is a major mechanism of horizontal gene transfer (HGT) and has huge implications for genome plasticity (2). Natural transformation can enable bacteria to acquire new traits, including drug resistance (3, 4), virulence (5), and metabolic functions (6), and plays a key role in nutrient acquisition and bacterial adaptation, as well as evolution and speciation (7–9). Therefore, a thorough understanding of the regulation for bacterial natural transformation can lead to discovering new targets of antibacterial action and reduce the emergence of drug-resistant bacteria or new pathogens.

The phenomenon of natural transformation was first discovered in Streptococcus pneumoniae (10), and in 1944, Avery et al. (11) first proved that DNA is the carrier of genetic information via the natural transformation of S. pneumoniae. S. pneumoniae is a commonly encountered Gram-positive bacterial pathogen, and infections can result in lobar pneumonia, tracheitis, otitis media, meningitis, and septicemia (12–14). Approximately 1.6 million people succumb to these infections each year (15). These deaths include 43.8% to 62.5% that are children under 5 years old and occur mostly in developing countries (16). S. pneumoniae drug resistance is also serious, and this pathogen was included in the list of antibiotic-resistant “key pathogens” published by the World Health Organization (WHO) in 2017. The acquisition of antibiotic resistance genes in S. pneumoniae is primarily due to natural transformation (17, 18), so S. pneumoniae was used as a model bacterium for studying the molecular mechanism of natural transformation.

Competence is a prerequisite of natural transformation in S. pneumoniae, in which the *comCDE* operon is the core regulator of competence. This pathogenic bacterium controls competence development through an elaborate two-layer regulation of basal and autoregulatory transcription of the *comCDE* (19). The competence-stimulating peptide (CSP) precursor is exported, and the signal peptide is cleaved by ComAB, allowing it to bind to ComD on the cell membrane and resulting in a ComD-activating autophosphorylation (20, 21). This protein then phosphorylates ComE, which activates its transcription factor activity for early competence gene expression, such as *com*X and *comCDE* (22, 23). ComX induces expression of at least 80 late competence genes, including *ssbB*, *recA*, and *dprA*, which encode single-stranded DNA (ssDNA)-binding proteins, homologous recombinant proteins, and DNA processing proteins, respectively; finally, the transformation is induced (24–26). Phosphorylated ComE can also participate in a positive feedback cycle by inducing the autoregulatory transcription of the *comCDE* operon, resulting in a rapid and transient high level of ComE protein expression, and making the transformation efficiency reach a peak quickly that generally occurs 5 to 10 min after CSP induction (27–30). Following this rapid activation, the transcription of *comCDE* decreases rapidly and returns to basal levels after 30 min, competence closes, and transformation can no longer occur (27). Interestingly, only ComE has been reported to directly regulate *comCDE* transcription in S. pneumoniae.

CcpA, a LacI/GalR protein family member, is a carbon catabolite regulator and can bind to *cis*-regulated catabolite response elements (cre) to activate or repress the transcription of target genes (31, 32). CcpA has been identified as a regulator of competence in Streptococcus gordoni, and its inactivation reduces the transcription of competence genes such as *comC*, *comD*, and *comYA* (33). CcpA in Streptococcus oligofermentum regulates competence development via a tRNA^Arg^ transcriptional read-through (34). In Streptococcus mutans, *ccpA* deletion mutants have slightly increased *com*X promoter activity when grown on glucose and significantly decreased activity when grown on fructose or trehalose (catabolite repression) (35). In S. pneumoniae, CcpA has been linked to carbohydrate metabolism, growth and reproduction, biofilm formation, and virulence regulation (36–38). In addition, previous microarray data have shown that *comE* is downregulated after *ccpA* depletion (36); however, whether *ccpA* is associated with competence in S. pneumoniae remains unclear.

Although the regulatory mechanisms of competence have been extensively studied in S. pneumoniae, the molecules that directly regulate *comCDE* transcription are poorly understood. In the current study, we confirmed that CcpA is involved in the regulation of competence and transformation of S. pneumoniae D39 by directly regulating *comCDE* transcription. RNA-sequencing (RNA-seq) results revealed that the deletion of *ccpA* was accompanied by a decreased transcription of competence genes involved in DNA uptake and recombination. We also found four atypical cre sites on P*comCDE*, and CcpA binds to these sites to comprehensively regulate *comCDE* transcription, ultimately resulting in the regulation of competence by CcpA in an optimal concentration-dependent manner. In addition, the carbohydrate (glucose and galactose) metabolism also plays a role in the CcpA-*comCDE* axis.

## RESULTS

### Deletion of *ccpA* inhibits the transformation of competent S. pneumoniae D39.

**FIG 1:**
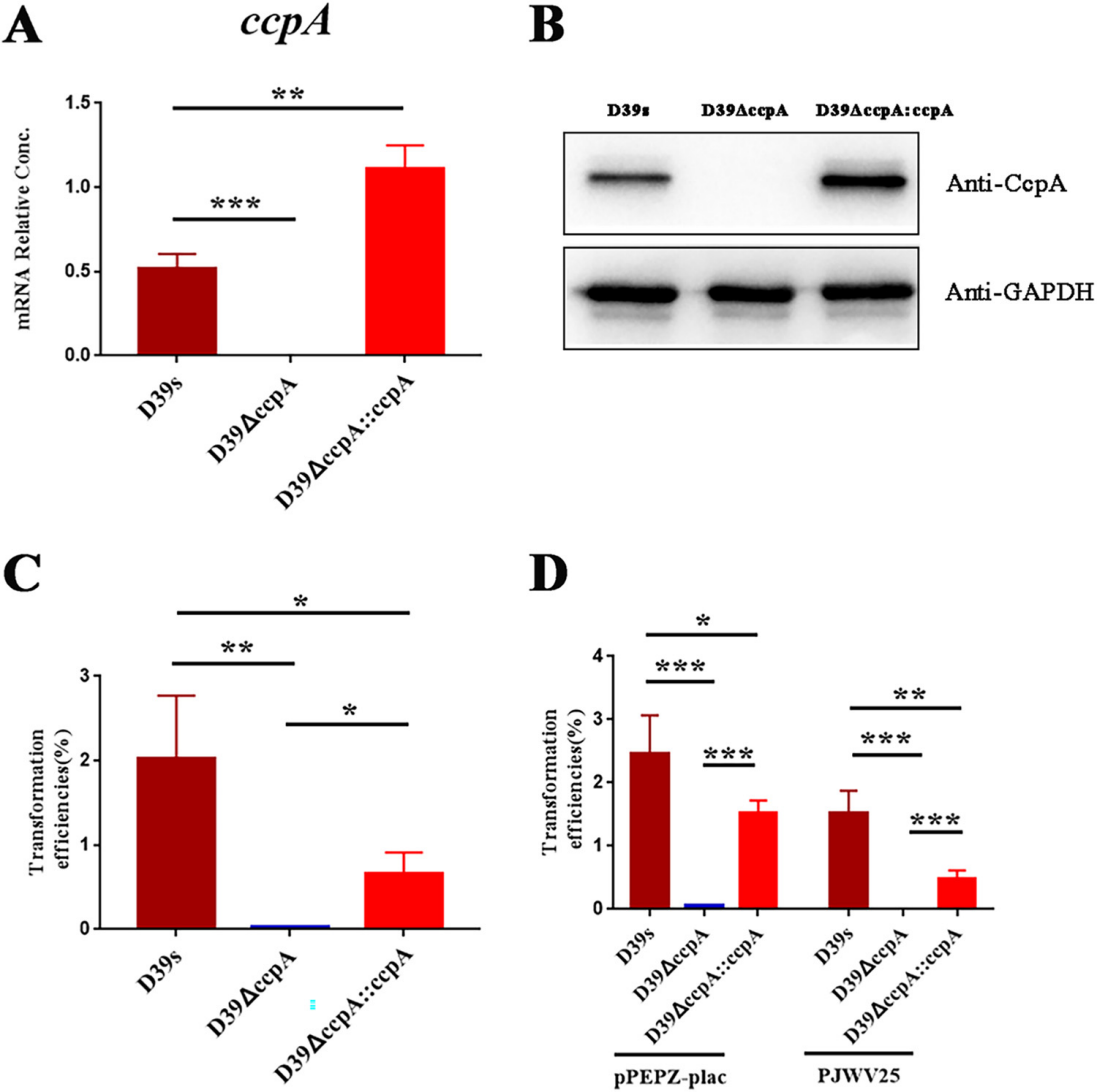
Effects of *ccpA* on S. pneumoniae transformation. (A) The *ccpA* mRNA of strains D39s, D39Δ*ccpA*, and D39Δ*ccpA*::*ccpA* were determined by real-time quantitative PCR (qPCR). qPCR was conducted in triplicate. (B) Use of Western blot to probe the expression of CcpA in D39s, D39Δ*ccpA*, and D39Δ*ccpA*::*ccpA*. A representative result for three independent experiments is displayed. (C, D) Effects of *ccpA* on the transformation of encapsulated S. pneumoniae using genomic DNA (gDNA) encoding an erythromycin resistance gene (C) and plasmids as DNA donors (D). Three replicates, averages, and standard errors of the mean (SEMs) are plotted. *, *P* < 0.05; **, *P* < 0.01; ***, *P* < 0.001. GAPDH, glyceraldehyde-3-phosphate dehydrogenase.

CcpA is involved in the control of competence and transformation regulatory networks in several streptococcal species (33–35). However, the regulatory role of CcpA in S. pneumoniae competence and transformation has not been reported. We used the Janus cassette (JC) (39) as a screening tool to construct *ccpA* silent mutations (D39Δ*ccpA*) in the streptomycin-resistant D39-derived strain (D39s) using a two-step transformation procedure. Compared with the traditional antibiotic substitution method, JC replacement can eliminate the polarization effect caused by the introduction of antibiotic resistance genes (39). The complete *ccpA* gene sequence was inserted into the shuttle plasmid pIB166 containing the PC promoter (40) and transferred into *ccpA*-deficient strain (D39Δ*ccpA*) to construct a *ccpA* complemented strain (D39 Δ*ccpA:ccpA*). In D39Δccp*A* strain, the *ccpA* mRNA and protein were not expressed. The *ccpA* complemented strain produced *ccpA* mRNA and protein levels that exceeded the parental strain D39s (i.e., a wild type) (Fig. 1A and B). These results indicated that *ccpA*-deficient and *ccpA*-complementary strains were successfully constructed.

Strains D39s, D39Δccp*A*, and D39Δccp*A*:ccp*A* were examined for their transformation efficiency using genomic DNA containing an erythromycin resistance marker, as well as plasmids pPEPZ-plac and PJWV25 as DNA donors. The erythromycin cassette is inserted into the D39s genome near SPD_1795. The pPEPZ-Plac and PJWV25 vectors (containing spectinomycin and tetracycline markers, respectively) could be integrated into D39s chromosome near SPD_1736 and bgaA, respectively (41, 42). Transformations efficiency of strain D39Δ*ccpA* were significantly reduced for both genomic DNA (gDNA) and plasmid DNA compared to wild type, while the complemented strain Δ*ccpA*::*ccpA* displayed a higher transformation efficiency (Fig. 1C and D). These results confirmed the role of CcpA in the activation of transformation.

### Optimal concentration effect of CcpA on transformation.

Studies have shown that transformation is regulated by multiple factors, such as growth rate (43) and capsule (41, 44). We observed that the *ccpA*-deficient strain showed growth inhibition (Fig. S1A and B) and capsule reduction (Fig. S1F and G). To assess whether perturbing growth rate and capsule could be related to the transformation in the *ccpA*-deficient strain, the pH of the C+Y medium was adjusted to 7.0, the level at which S. pneumoniae competence is inhibited (Fig. S1C) (45). Under this condition, the Δ*ccpA* strain still displayed growth defects in the exponential growth phase before and after CSP treatment (Fig. S1D) that were similar to its growth at pH 8.0 (permissive for natural competence initiation) (Fig. S1E). Additionally, an unencapsulated mutant (D39Δ*cps*) was tested, and its transformation efficiency was also significantly reduced after *ccpA* deletion (D39Δ*cpsΔccpA*) (Fig. S1H). These results indicated that the growth delay and capsule production of the Δ*ccpA* mutants were independent of competence regulation.

**FIG 2:**
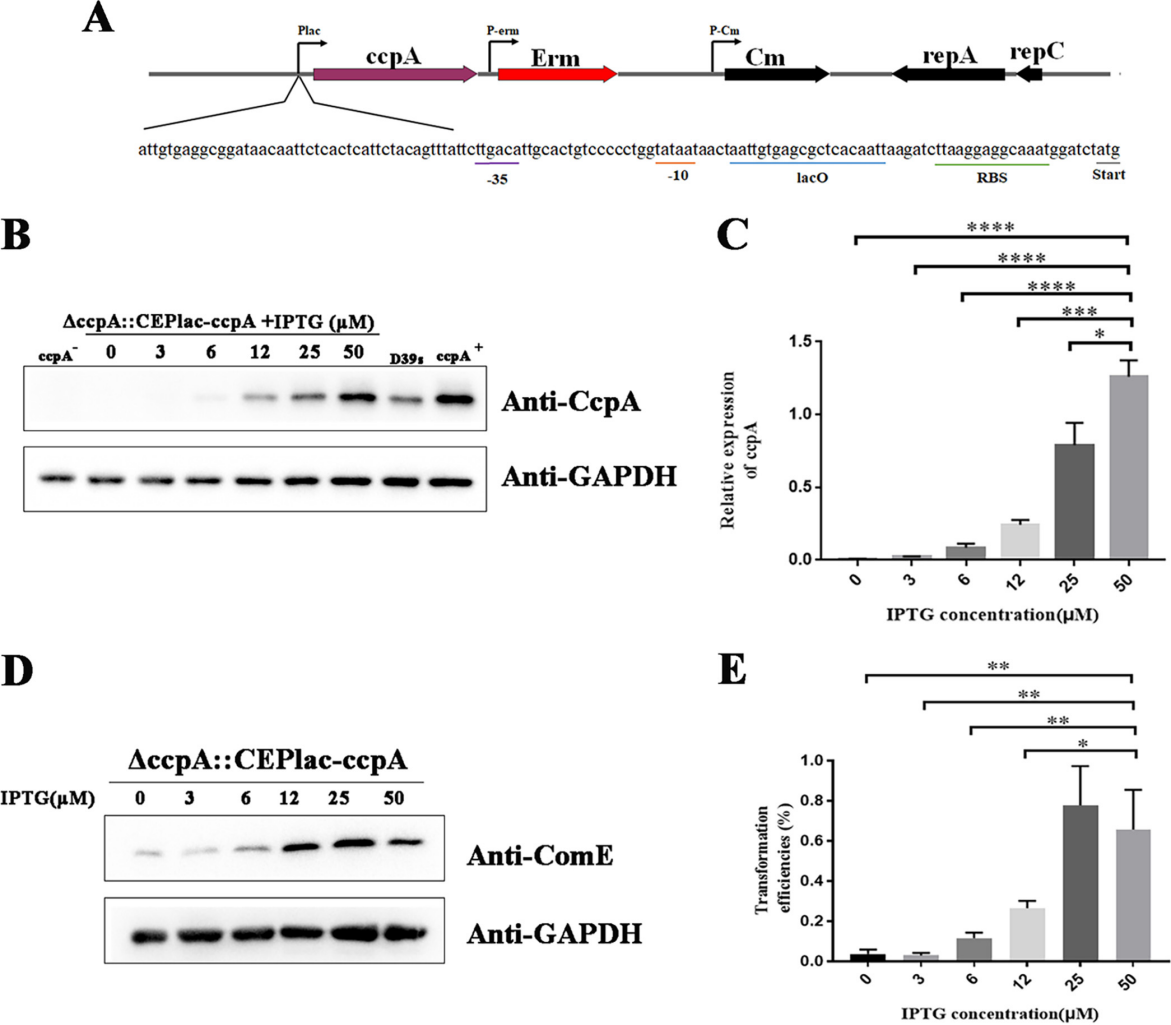
Dosage effects of CcpA on transformation. (A) Genetic organization of CEPlac-ccpA expression platform. The Cm gene is constitutively expressed via the P-_Cm_ promoter and confers chloramphenicol resistance. The *ccpA* gene is expressed via Plac, the sequence of which is shown. (B) Western blot comparing expression levels of CEP*lac-dprA* in various isopropyl β-d-1-thiogalactoside (IPTG) concentrations to those of wild-type, Δ*ccpA* (*ccpA*^–^), and Δ*ccpA*::*ccpA* (*ccpA*^+^) cells. (C) qPCR measurements of *ccpA* mRNA levels from strain Δ*ccpA*::CEPlac-*ccpA* at the indicated IPTG concentrations. (D) Western blot comparing ComE expression levels of Δ*ccpA*::CEPlac-*ccpA* strain at the indicated IPTG concentrations. (E) Transformation efficiencies of Δ*ccpA*::CEPlac-*ccpA* at the indicated IPTG concentrations. All strains were grown in C+Y medium containing different concentrations of IPTG. At an optical density at 600 nm (OD_600_) of ~0.10, CSP was treated for 10 min, and then bacterial precipitate was collected for Western blotting (WB) or qPCR. Average of three replicates and SEMs are plotted. *, *P* < 0.05; **, *P* < 0.01; ***, *P* < 0.001; ****, *P* < 0.0001. RBS, ribosome-binding site; Start, start codon (ATG).

Here in this study, we hypothesized that *ccpA* could directly regulate transformation. To further explore the role of *ccpA* in transformation, we constructed a chromatin expression platform (CEP) using the shuttle plasmid pIB166, which allows *ccpA* expression to be controlled (46). A complemented strain, Δ*ccpA*::CEPlac-*ccpA*, was constructed in a D39Δ*ccpA*-lacI (D39Δ*ccpA* derivative carrying *lacI* gene) background, in which *ccp*A was under the control of the Plac promoter (Fig. 2A). The autoinducer isopropyl β-d-1-thiogalactoside (IPTG) controls the transcription of Plac promoter. Previous studies of IPTG addition to S. pneumoniae cells indicated that it does not induce transcriptional alterations (47), so that any observed effects in our constructed strain would be due solely to alterations in cellular CcpA levels. Both *ccpA* mRNA and protein in strain Δ*ccpA*::CEPlac-*ccpA* increased in an IPTG concentration-dependent manner (Fig. 2B and C). The transformation efficiency of strain Δ*ccpA*::CEPlac-*ccpA* increased in an IPTG concentration-dependent manner when the IPTG was <25 μM (Fig. 2E), and at this level ComE also increased (Fig. 2D). However, at an IPTG level of 50 μM, transformation efficiencies were not further increased (Fig. 2E) and ComE protein levels were slightly reduced (Fig. 2D). We observed that *ccpA*-complement strains had higher CcpA protein levels than strains with 50 μM IPTG levels (Fig. 2B). However, compared with wild type (WT), the transformation efficiency of *ccpA*-complement strains was reduced (Fig. 1C and D). These results demonstrated that a critical level of CcpA is required to ensure optimal transformation of S. pneumoniae.

### CcpA affects *comCDE* transcription in the early exponential growth stage.

**FIG 3:**
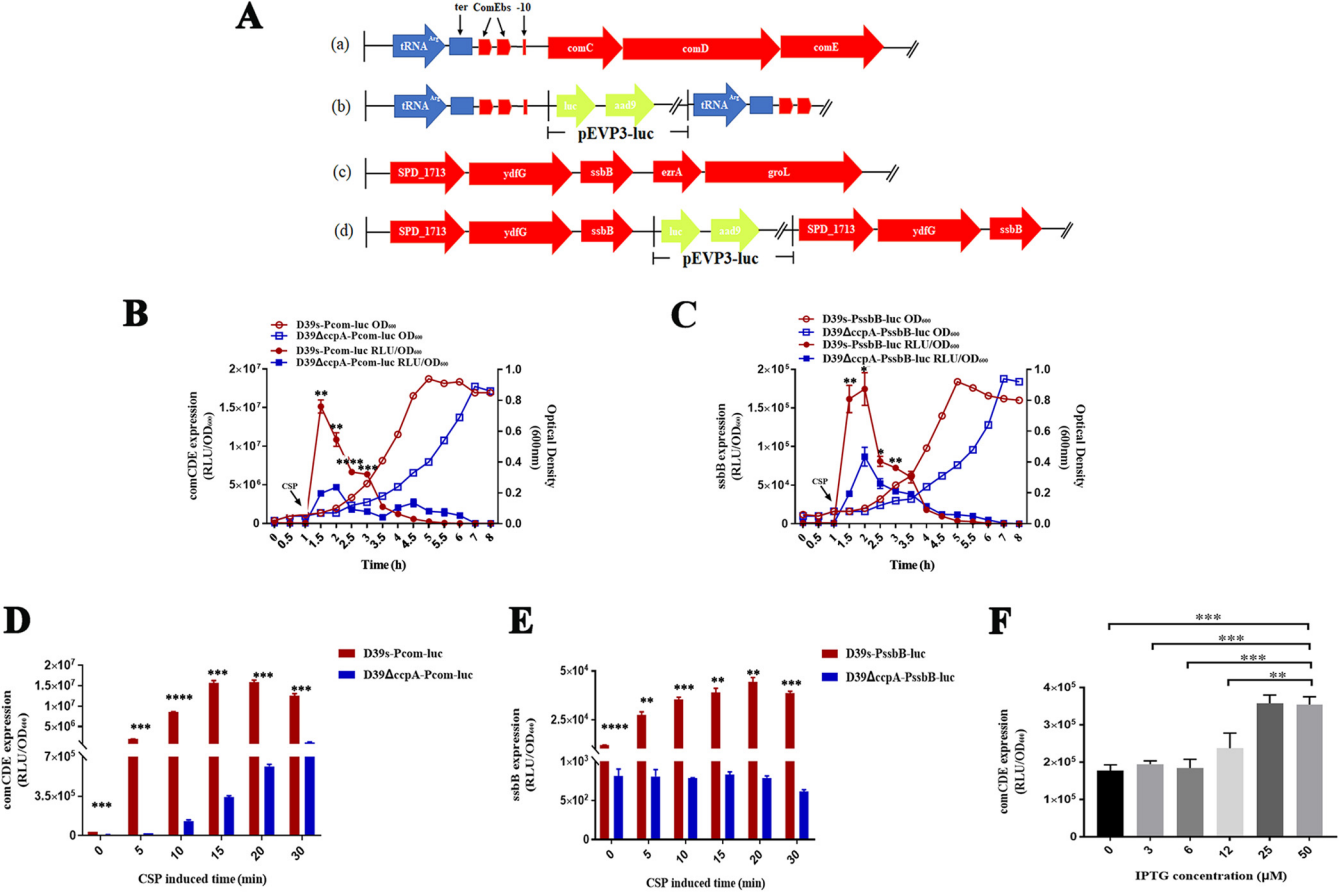
Transcription of *comCDE* and *ssbB* in S. pneumoniae. (A) (Panel a) Diagram of the genomic organization of tRNA^Arg^-comCDE in a wild-type strain. (Panel b) Construction of the luciferase reporter strain for *comCDE* expression. (Panel c) Diagrams of the genomic organization of *ssbB* in a wild-type strain. (Panel d) Construction of the luciferase reporter strain for *ssbB* gene expression. (B) Transcription profiles of the *comCDE* operon in wild-type strain and *ccpA* deletion mutant during growth were determined using luciferase reporter strains WT-Pcom-luc and ΔccpA-Pcom-luc. *comCDE* transcription was reported by luciferase activity (relative light units [RLU] per OD_600_) of WT-Pcom-luc (red solid lines) and D39ΔccpA-Pcom-luc strains (blue solid line), respectively. Overnight cultures of the luciferase reporter strains were each diluted into fresh C+Y medium and cultured at 37°C. Luciferase activity and optical density at 600 nm (OD_600_) were measured at 30-min intervals. *comCDE* is expressed as RLU per OD_600_. (C) Luciferase activities and growth curves of the WT-PssbB-luc (red line with circles) or D39ΔccpA-PssbB-luc (blue line with squares) strains. Luciferase activity and OD_600_ were measured at 30-min intervals. *ssbB* is expressed as RLU per OD_600_. (D, E) Transcription profiles of *comCDE* (D) and *ssbB* (E) during the early exponential growth in wild-type and the *ccpA* deletion mutant. At an OD_600_ of ~0.10, luciferase activity and OD_600_ were measured at 10-min intervals. The *comCDE* and *ssbB* expressions are expressed as RLU/OD_600_. (F) The luciferase gene was fused downstream of P*comCDE* of Δ*ccpA*::CEPlac-*ccpA* strain to measure *comCDE* transcription. All strains were grown in C+Y medium. At an OD_600_ of ~0.10, CSP was treated for 10 min, and then luciferase activity was measured. *, *P* < 0.05; **, *P* < 0.01; ***, *P* < 0.001; ****, *P* < 0.0001.

The *comCDE* operon is the core regulator of competence and transformation in S. pneumoniae. To test whether CcpA regulates transformation by affecting *comCDE* transcription, luciferase reporter strains D39s-Pcom-luc and Δ*ccpA*-Pcom-luc were constructed by fusing the luciferase gene with the P*comCDE* to monitor the transcription profiles of the *comCDE* in wild-type strain and Δ*ccpA* mutant during growth (Fig. 3A). SsbB is a late competence gene that generates a reservoir of DNA for transformation, facilitating multiple transformation events (48). Therefore, the luciferase gene was fused to the C terminus of the *ssbB* gene to construct the *ssbB* luciferase strain to track expression of late competence genes (Fig. 3A). Luciferase activity of *comCDE* increased in both strains upon addition of CSP, and the transcription level of Δ*ccpA* mutant was significantly lower than the wild-type strain at the early exponential growth stage (Fig. 3B). Consistent with *comCDE* transcription, *ssbB* luciferase activity was increased after the addition of CSP and significantly decreased in Δ*ccpA* mutants during the early exponential growth phase compared with the wild type (Fig. 3C).

Since the *comCDE* and *ssbB* transcriptions in the wild-type strain and Δ*ccpA* mutants were significantly induced in the early exponential growth phase after the addition of CSP, we further examined the transcription of *comCDE* and *ssbB* at six time points in the early exponential growth phase. Transcription of *comCDE* and *ssbB* in the Δ*ccpA* mutant were significantly less than the wild-type strain (Fig. 3D and E). These results strongly suggested that *ccpA* directly promotes competence in the early exponential growth stage.

A luciferase gene fusion was constructed downstream of P*comCDE* in strain Δ*ccpA*::CEPlac-*ccpA* to monitor *comCDE* transcription in the early exponential growth stage. Luciferase activity was also increased in an IPTG concentration-dependent manner at IPTG < 25 μM but no longer increased at 50 μM (Fig. 3F) and paralleled with the transformation efficiency (Fig. 2E). Altogether, these results strongly suggested that competence induction of S. pneumoniae requires a critical level of CcpA to ensure optimal *comCDE* transcription and transformation.

### *ccpA* deletion alters the dynamic developmental pattern of competence.

**FIG 4:**
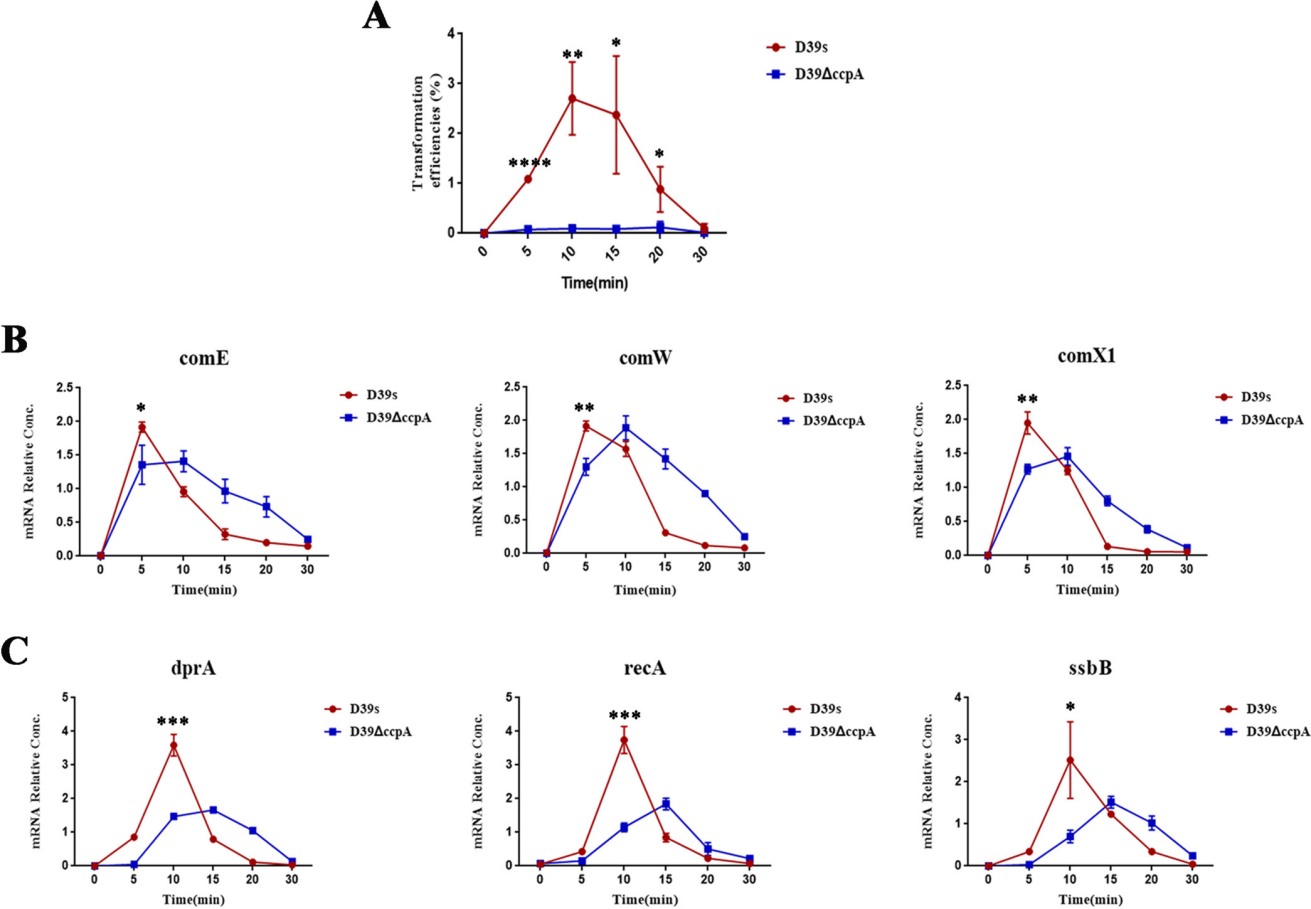
Kinetics of transformation efficiency and gene expression during competence. (A) Transformation efficiency at six time points immediately before or after exposure to exogenous CSP in strains D39s and Δ*ccpA*. The D39s and Δ*ccpA* strains were grown on C+Y medium. At an OD_600_ of ~0.10, CSP was added, and the transformation efficiency was measured at the indicated time points after CSP addition. (B) The relative abundance of early competence genes with different CSP treatment times. (C) The relative abundance of late competence genes with different CSP treatment times. *, *P* < 0.05; **, *P* < 0.01; ***, *P* < 0.001; ****, *P* < 0.0001.

To further clarify the effect of CcpA on *comCDE* transcription of S. pneumoniae, competence development was examined at six time points in the wild-type and *ccpA*-deficient strains. In the early exponential growth stage, S. pneumoniae D39 showed a typical competence development pattern, and transformation efficiency reached the maximum at 10 min following CSP treatment. After CSP treatment for 30 min, competence was closed, and no transformation was observed. In the Δ*ccpA* mutant, the transformation efficiency also increased after CSP induction, but their peak times and levels were significantly lower than the wild type (Fig. 4A).

Activation of the competence pathway leads to increased expression of 5% to 10% of the pneumococcal genome in two main waves of gene expression (27, 49). The first wave of induction is carried out directly by ComE on a subset of early genes that include *comAB*, *comCDE*, and the alternate sigma factor *com*X. The second wave of competence induction is regulated by ComX that leads to an increase in the expressions of at least 80 late genes (19, 50), and 14 have been identified as essential for transformation (51). Intracellular ssDNA will be sequestered by SsbB and will first be replaced by the DprA, which will load it onto RecA in a process that requires both CoiA and RadA for incorporation into the chromosome through homologous recombination (52, 53). Both DprA and RecA protect transforming ssDNA from degradation (54). Once RecA is loaded onto ssDNA, it polymerizes and promotes chromosomal integration by homologous recombination. Early and late competence gene expression, including several genes in the above system, was quantified at six time points immediately before or after exposure to exogenous CSP to examine whether *ccpA* affects the expression of competence genes. Immediately upon CSP addition, early competence genes *comE*, *comW*, and *comX1* and late competence genes *recA*, *ssbB*, and *dprA* of the wild-type strain were rapidly induced, reaching peak transcriptional levels at 5 and 10 min, respectively, followed by a rapid decay at 30 min (Fig. 4B and C). In the wild-type strain, DNA uptake and recombination-related genes (*recA*, *ssbB*, and *dprA*) were significantly increased at 10 min (Fig. 4C), which is the point at which transformation efficiency was also maximal (Fig. 4A). The expression levels of *dprA*, *ssbB*, and *recA* decreased rapidly after this time. In the *ccpA* deletion mutant, expression of the early competence genes *comE*, *comW*, and *comX1* and late competence genes *recA*, *ssbB*, and *dprA* reached maximal levels at 10 and 15 min, respectively (Fig. 4B and C). The peak level of early competence genes in the *ccpA*-deficient bacteria was slightly lower than the wild type (Fig. 4B). However, the peak expression levels of late competent genes involved in DNA uptake and recombination-related genes (*recA*, *ssbB*, and *dprA*) were significantly lower than that of the wild type (Fig. 4C). This result was consistent with the significantly lower transformation efficiency of Δ*ccpA* compared with the wild type.

At 10 min of CSP treatment, the transformation efficiency and late competence gene expression were significantly different between wild-type and Δ*ccpA* mutant strains (Fig. 4A and C). At this stage, global gene expression profiling was undertaken by RNA-seq to further explore the mechanism of *ccpA*-regulated competence. Specifically, the expression profile of the Δ*ccpA* strain was compared to that of WT in normal cultures (C+Y medium). A total of 224 upregulated (*P* value < 0.05 and log_2_ fold change ≥ 1) and 239 downregulated genes (*P* value < 0.05 and log_2_ fold change ≤ −1) were identified (Fig. S2A). Except for the genes regulated by *comE* and *comX*, there were no significant changes in the other regulators of competence (|log_2_ fold change| ≥ 1.5) (Table S3). Genes involved in nucleotide metabolism were detected among those downregulated (Fig. S2C), particularly those competence genes related to DNA uptake and recombination (|log_2_ fold change| ≥ 1.5) (Table S3). The transcriptome results were consistent with the quantitative PCR (qPCR) results, especially that the late competence genes related to DNA uptake were downregulated after *ccpA* deletion, indicating that *ccpA* affected the dynamic developmental pattern of competence by regulating *comCDE* transcription, thereby regulating transformation.

### Identification of CcpA-binding sites in the *comCDE* promoter.

**FIG 5:**
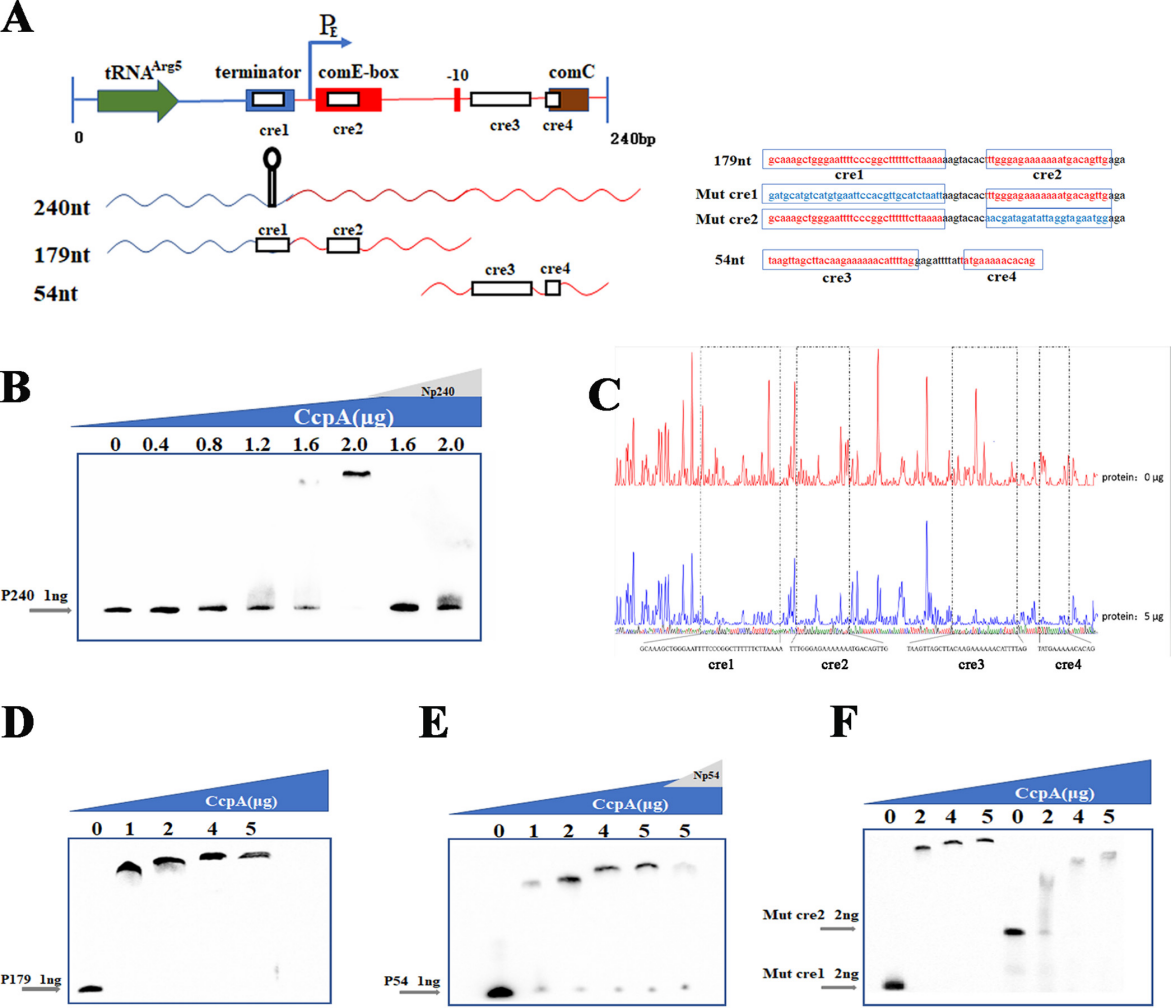
Electrophoretic mobility shift assay (EMSA) determines CcpA binding to the P*comCDE*. (A) Schematic diagram depicting tRNA^Arg^ and the downstream intergenic region between tRNA^Arg^ and P*comCDE*. (B) Binding of CcpA to the P240 fragment. Binding reactions to 1 ng P240 were conducted with CcpA protein concentrations from 0 to 2.0 μg (lanes 1 to 6), and 100 ng of non-biotin-labeled P240 (Np240) (lanes 7 and 8) were added to compete with the labeled probe. (C) DNase I footprinting assay. (D) Binding of CcpA to the P179 fragment. Binding reactions to 1 ng P179 were conducted with CcpA protein concentrations from 0 to 5 μg (lanes 1 to 5). (E) Binding of CcpA to the P54 fragment. Binding reactions to 1 ng P54 were conducted with CcpA protein concentrations from 0 to 5.0 μg (lanes 1 to 5), and 100 ng of non-biotin-labeled P54 (Np240) (lane 6) were added to compete with the labeled probe. (F) EMSA was conducted to determine CcpA binding to Mut cre1 (lanes 1 to 4) or Mut cre2 (lanes 5 to 8). Gray and black arrows specify the free probes and protein-DNA complexes, respectively. nt, nucleotide. Cre, catabolite response elements.

CcpA can bind to cre elements to activate or repress target gene transcription (31, 55). To gain insights into the molecular mechanism by which CcpA regulates S. pneumoniae competence, we first identified whether the cre is present in P*comCDE*. S. pneumoniae CcpA was overproduced in a heterologous system and purified to homogeneity (Fig. S3A). A 240-nucleotide (nt) DNA fragment consisting of the P*comCDE* was labeled with biotin to generate the DNA probe P240 (Fig. 5A). A DNA electrophoretic mobility shift assay (EMSA) was then performed with a mixture of 1 ng of P240 with CcpA protein at concentrations ranging from 0.4 to 2.0 μg. The promoter region of *comCDE* displayed reduced electrophoretic mobility with increasing concentrations of CcpA. Addition of a non-biotin-labeled P240 cold probe (Np240) to the binding mixture diminished the DNA-protein complex (lanes 7 and 8) (Fig. 5B). These data indicated that CcpA specifically binds to P*comCDE*. DNase I footprinting was then used to determine the exact DNA sequences to which CcpA bound. When the concentration of CcpA was 5 μg, 4 binding sequences appeared of lengths 36, 24, 30, and 14 nucleotides (Fig. 5C). To further determine the binding of CcpA to the 4 cre sequences, a 179 nt-DNA fragment containing cre1 and cre2 (P179), and a 54-nt DNA fragment containing cre3 and cre4 (P54) were synthesized and labeled at the 5′ end with biotin (Fig. 5A). Protein-P179 and protein-P54 DNA complexes were formed with a CcpA concentration of 1 μg (Fig. 5D), and the complex was diminished by the addition of a non-biotin-labeled P54 probe (Np54) (Fig. 5E). We then generated cre point mutant fragments (Mut cre1 and Mut cre2) that were then biotin labeled (Fig. 5A). Mut cre1 and Mut cre2 displayed reduced electrophoretic mobility with increasing concentrations of CcpA (Fig. 5F), indicating that CcpA binds to both cre1 and cre2. These results demonstrated that CcpA binds to P*comCDE* by recognizing cre sequences.

### CcpA regulates transformation by binding to the cre sequence on the *comCDE* promoter.

**FIG 6:**
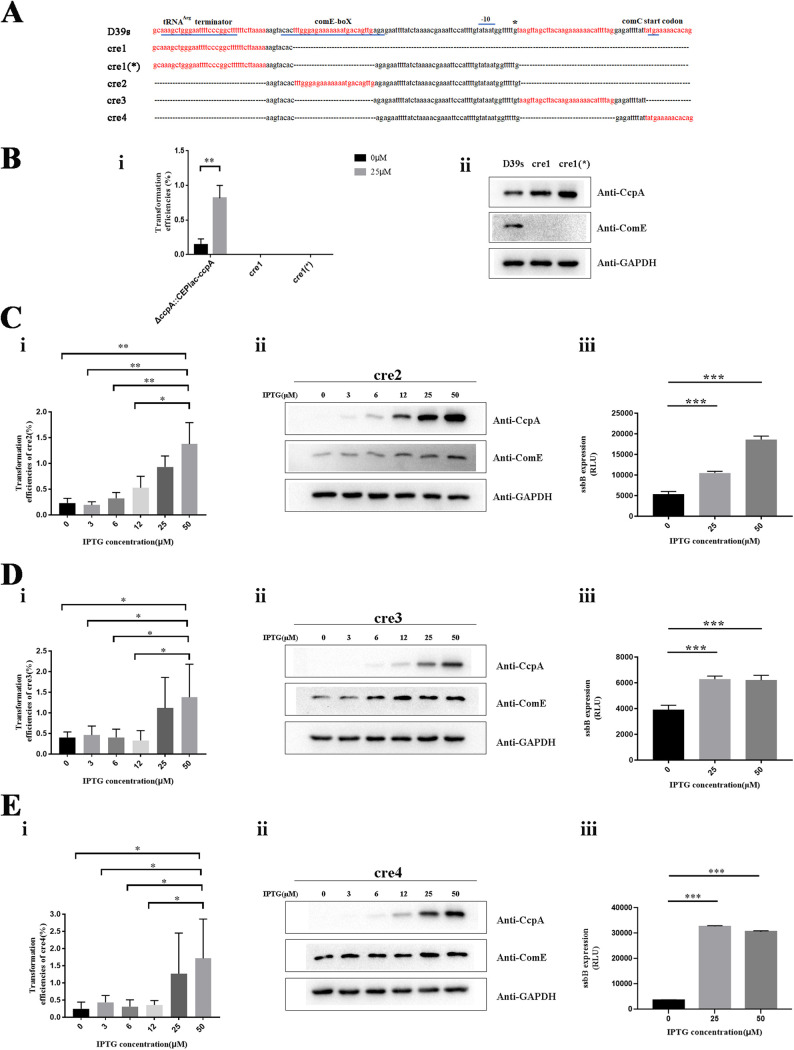
Effects of CcpA binding to cre at the P*comCDE* on S. pneumoniae D39 transformation. (A) Genetic organization of the P*comCDE* in different cre strains. The panel shows the DNA sequence of the tRNA^Arg^-comCDE region containing the tRNA^Arg^ terminator, ComE binding sites, the promoter region of *comCDE* (−10 and −35 sequences), and the start codon of comC gene. The bold red letters represent the four cre regions that CcpA binds to the P*comCDE*. D39s represents the genetic organization of the P*comCDE* in WT; cre1, cre1(*), cre2, cre3, and cre4 represent the genetic organization of the P*comCDE* in different cre strains. The dashed lines mean bases that are deleted. (B to E) The effects of cre1 (B), cre2 (C), cre3 (D), and cre4 (E) on S. pneumoniae transformation. (Panel i) Transformation efficiency. (Panel ii) Detection of CcpA and ComE expression by Western blotting. (Panel iii) Transcription profiles of the *ssbB*. A series of strains containing only single cre sequences in a strain Δ*ccpA*::CEPlac-*ccpA* background was constructed. All strains were grown in C+Y. At an OD_600_ of ~0.10, CSP was treated for 10 min, and then ComE protein and luciferase activity were measured. *, *P* < 0.05; **, *P* < 0.01; ***, *P* < 0.001. RLU, relative light units.

CcpA can activate or repress target gene transcription by binding to a typical consensus CcpA-binding site TGWAANCGNTNWCA (56). We compared the typical cre sequence with the cre sites on P*comCDE* and no matches were found (Fig. S4). Interestingly, CcpA-binding site architecture in Gram-positive bacteria is also highly variable in both length and base composition, i.e., atypical cre sites (57). Therefore, we hypothesized that CcpA may bind to the four atypical cre sites on P*comCDE* to regulate *comCDE* transcription. We constructed a series of strains containing only single cre sequences in a strain Δ*ccpA*::CEPlac-*ccpA* background (Fig. 6A). IPTG was added to the culture medium to control CcpA expression, and we compared the transformation efficiencies of these strains. cre1 is located upstream of the P*comCDE*, the tRNA^Arg^ gene transcription termination region (TRT) that regulates one-third of the *comCDE* basal transcription (19). Under CcpA expression conditions, strains retaining only cre1 (with or without the transcription start site) produced no transformants (Fig. 6B, panel i), nor was ComE protein expressed (Fig. 6B, panel ii). These results suggested that cre1 alone cannot mediate CcpA regulation of transformation. cre2 is located in the comE box region, and phosphorylated and dephosphorylated ComE can regulate the *comCDE* transcription by competing for binding to the comE box (58). The transformation efficiency of the cre2 strain was lower when CcpA was not expressed, but with the increase of IPTG concentration, CcpA was induced; the transformation efficiency of the cre2 strain increased significantly (Fig. 6C, panel i), and the ComE protein levels also increased significantly (Fig. 6C, panel ii). CcpA can therefore regulate transformation by binding to the cre2 site in a concentration-dependent manner. cre3 is located after the transcription start site, and cre4 is located in the *comC* coding region. The cre3 and cre4 strains displayed no differences in transformation efficiency for IPTG at 0 to 12 μM, but at 25 μM, their transformation efficiencies increased significantly (Fig. 6D, panel i, and 6E, panel i), and ComE protein levels also increased (Fig. 6D, panel ii and 6E, panel ii). However, at 50 μM IPTG, neither the transformation efficiency nor ComE protein levels increased (Fig. 6D and E), which was consistent with the previous optimal concentration effect of CcpA (Fig. 2).

*ssbB* is a late competence gene related to DNA uptake and recombination, so we measured *ssbB* transcription to track expression of late competence genes. In cre2 strains, *ssbB* transcription increased in a IPTG concentration-dependent manner (Fig. 6C, panel iii). In cre3 and cre4 strains, the transcriptional activity of *ssbB* were maximal at 25 μM IPTG (Fig. 6D, panel iii, and 6E, panel iii). Therefore, cre2 strains can promote transformation in a CcpA concentration-dependent manner, and both cre3 and cre4 strains achieved optimal transformation levels at 25 μM IPTG.

### CcpA and ComE coregulate the transcription of *comCDE* operon.

**FIG 7:**
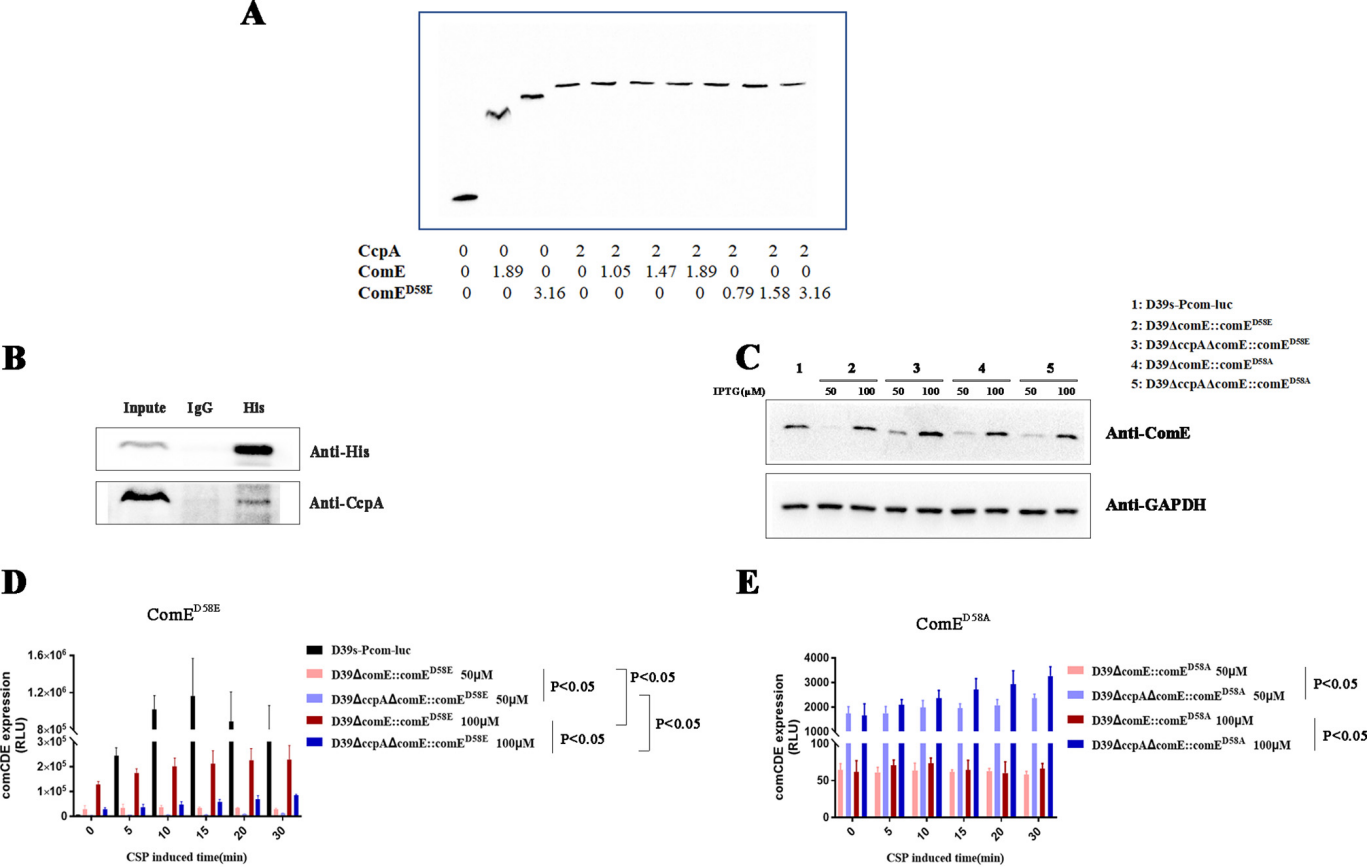
Regulation of *comCDE* transcription by CcpA, ComE^D58E^, or ComE^D58A^. (A) Competitive EMSA was conducted to determine CcpA, wild-type ComE, and ComE^D58E^ binding to the P*comCDE*. (B) Interaction of CcpA with ComE was verified by COIP. (C) ComE protein of ComE^D58E^ and ComE^D58A^ mutants were determined by Western blotting. All strains were grown in C+Y medium containing different concentrations of IPTG. At an OD_600_ of ~0.10, CSP was treated for 10 min, and then bacterial precipitate was collected by centrifugation for WB. (D, E) Transcription profiles of *comCDE* of ComE^D58E^ (D) and ComE^D58A^ mutants (E). At an OD_600_ of ~0.10, luciferase activity was measured at the indicated time points. The *comCDE* expression was expressed as RLU because luciferase was significantly different among the groups, and bacterial growth (OD_600_) over a short period of time did not change the results. RLU, relative light units.

We confirmed that cre2, the binding site of CcpA on the *PcomCDE*, is located in the ComE binding region (comE box). This suggests that CcpA and ComE may compete or synergistically combine P*comCDE* to regulate competence development. First, we identified whether CcpA and ComE had competitive binding on P*comCDE* by competitive EMSA. S. pneumoniae ComE (Fig. S3B) and ComE^D58E^ (Fig. S3C) proteins were then overexpressed in a heterologous system and purified to homogeneity. The binding of CcpA, ComE, and ComE^D58E^(simulate aspartic acid phosphorylation of ComE) on the P*comCDE* was analyzed using competitive EMSA. There was no significant change in the amount of protein-DNA complex in the presence of two proteins alone or at the same time, indicating that there was no competition between CcpA and ComE on P*comCDE* (Fig. 7A). Therefore, we tested whether there was an interaction between CcpA and ComE by coimmunoprecipitation. The *comE* gene from D39s was tagged with a 6×His tag and then fused with pPEPZ-Plac plasmid to construct plasmid pPEPZ-ComE (His). Plasmid pPEPZ-ComE (His) was transferred into D39s (containing lacI), and ComE (His) was overexpressed by the addition of IPTG to the medium. Then ComE (His) was enriched on magnetic beads. Finally, Western blotting (WB) was used to verify whether there was an interaction between CcpA and ComE. The results confirmed that CcpA interacted with ComE (Fig. 7B), suggesting that CcpA may coordinate with ComE on P*comCDE*.

The ComE protein bound to the comE box region has two forms: phosphorylated and dephosphorylated. CcpA promotes transformation by binding to cre2, and CcpA may therefore cooperate with phosphorylated and dephosphorylated ComE to regulate the *comCDE* transcription. To document this role, we generated a ComE^D58E^ mutant protein to simulate aspartic acid phosphorylation and ComE^D58A^ to simulate aspartic acid dephosphorylation (22). ComE^D58E^ and ComE^D58A^ were fused with pPEPZ-Plac to construct plasmids pPEPZ-ComE^D58E^ and pPEPZ-ComE^D58A^. This placed the expression of ComE^D58E^ or ComE^D58A^ under the control of an IPTG-inducible promoter. These were then expressed in a comE null mutant (carrying lacI gene) to exclude the effects of wild-type ComE phosphorylation and dephosphorylation, and luciferase activity was measured. The ComE protein in both strains increased in an IPTG concentration-dependent manner (50 and 100 μM, respectively) (Fig. 7C). When ComE^D58E^ was expressed, the *comCDE* transcription of the D39Δ*comE*::*comE*^D58E^ and D39Δ*ccpAΔcomE*::*comE*^D58E^ strains was enhanced with the increase of ComE^D58E^ protein concentration. However, the deletion of *ccpA* in strain D39Δ*ccpAΔcomE*::*comE*^D58E^ resulted in lower *comCDE* transcription than for strain D39ΔcomE::comE^D58E^(Fig. 7D). This result indicated that CcpA can promote the activation of *comCDE* transcription by ComE^D58E^. The *comCDE* transcription of the ComE^D58A^ mutant was significantly lower than that of the ComE^D58E^ mutant and did not change with the increase of ComE^D58A^ protein concentration (Fig. 7D and E). In contrast to the ComE^D58E^ mutant, *comCDE* transcription after *ccpA* deletion in strain D39Δ*ccpAΔcomE*::*comE*^D58A^ was significantly higher than for D39ΔcomE::comE^D58A^ (Fig. 7E). ComE^D58A^ has an inhibitory effect on the *comCDE* transcription, but the inhibitory effect was more obvious in the presence of CcpA, indicating that CcpA enhanced the inhibitory effect of ComE^D58A^ on *comCDE* transcription. These results suggested that CcpA could coregulate with phosphorylated and dephosphorylated ComE.

We also found that when IPTG concentration was at 100 μM, the ComE protein level was higher than that of wild type (D39s-Pcom-luc) (Fig. 7C), but the *comCDE* transcription of ComE^D58E^ mutant was significantly lower than that of wild type (Fig. 7D), and the transformation efficiency was also significantly lower (ComE^D58E^ mutants produced few or no positive transformants). This result suggested that phosphorylated ComE and dephosphorylated ComE coordinate to regulate the development of the optimal competence.

### Carbohydrate affects CcpA regulation of *comCDE* transcription.

**FIG 8:**
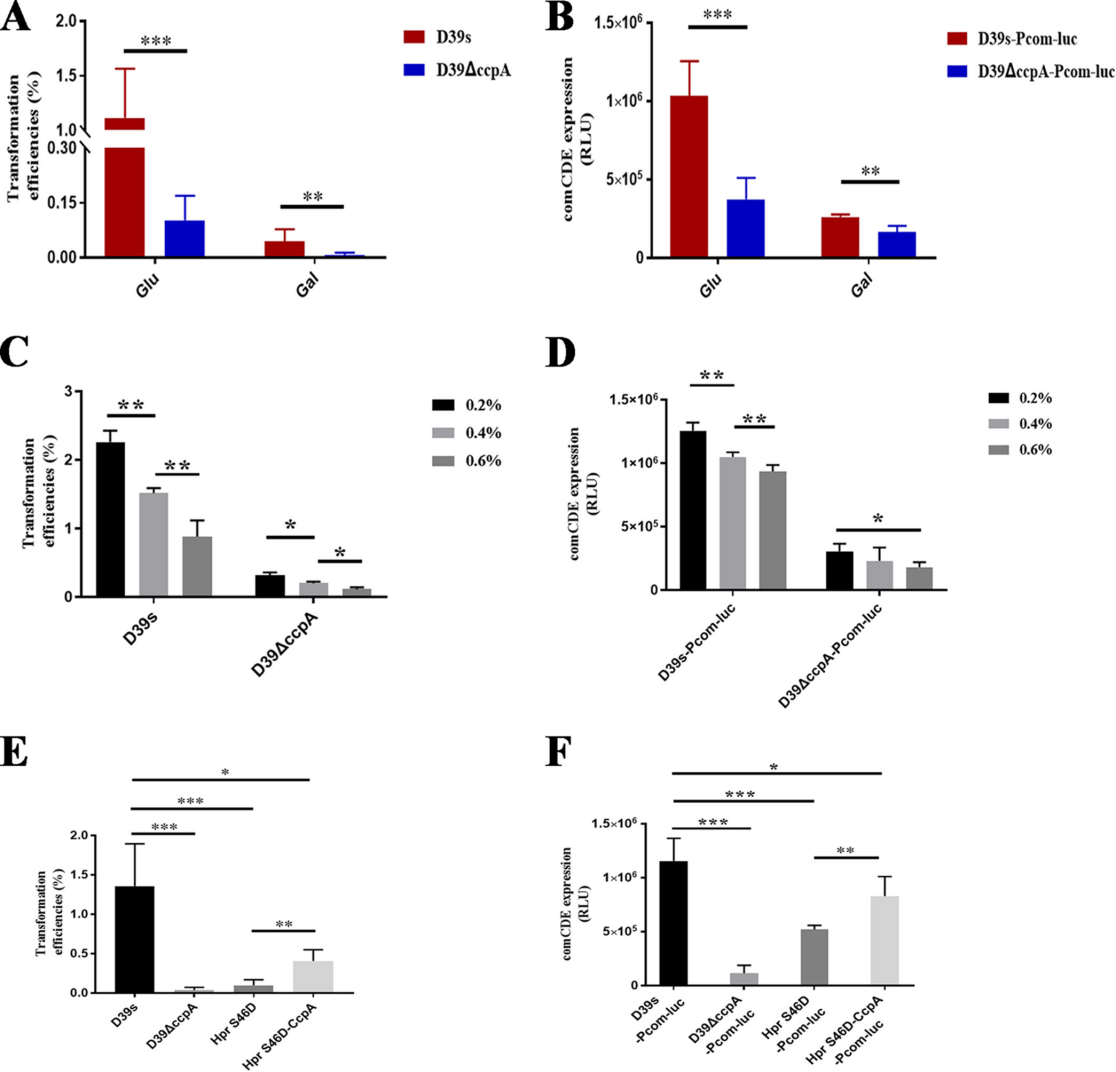
Effects of carbohydrates on CcpA regulatory activity. (A) Transformation efficiencies of D39s and D39ΔccpA in fresh C+Y medium containing either 0.2% glucose (Glu) or 0.2% galactose (Gal). (B) Effects of Glu and Gal on *comCDE* transcription. (C) Transformation efficiencies of D39s and D39ΔccpA strains in fresh C+Y medium containing different concentrations of glucose. (D) *comCDE* transcription in D39s-Pcom-luc and D39ΔccpA-Pcom-luc strains in fresh C+Y medium containing different concentrations of glucose. (E) Transformation efficiencies of D39s, D39ΔccpA, HPr S46D, and HPr S46D-CcpA strains in fresh C+Y medium. (F) *comCDE* transcription of D39s, D39ΔccpA, HPr S46D, and HPr S46D-CcpA strains in fresh C+Y medium. When the strains were grown in C+Y to an OD_600_ of ~0.10, CSP was treated for 10 min, and then luciferase activity was measured. The *comCDE* expression was expressed as RLU because luciferase was significantly different among the groups, and bacterial growth (OD_600_) over a short period of time did not change the results. *, *P* < 0.05; **, *P* < 0.01; ***, *P* < 0.001. RLU, relative light units.

CcpA acts as a global regulator of carbohydrate metabolism genes, and our RNA-seq results indicated that these genes were upregulated in the Δ*ccpA* strain (Fig. S2B), particularly sugar-specific enzymes II genes associated with transporting nonpreferred carbohydrates via the phosphoenolpyruvate-dependent phosphotransferase system (PTS) (|log_2_ fold change| ≥ 1.5) (Table S4). Sugar-specific enzyme II is a PTS component that mediates the internalization of extracellular carbohydrates. CcpA-mediated regulation depends on the concentration of carbohydrates and sugar metabolites (such as Fructose1, 6-diphosphate (FDP)) that can stabilize the protein complex with cre (59, 60). Therefore, we hypothesized that carbohydrates may affect the transcriptional regulation of CcpA, thereby affecting *comCDE* transcriptional and transformation. Accordingly, we examined the transformation efficiencies of S. pneumoniae grown in the presence of glucose and galactose that are prevalent in many niches occupied by pneumococci (61). A higher transformation efficiency was observed in glucose than in galactose-grown cells (Fig. 8A). Luciferase activity indicated that the *comCDE* transcriptional activity was consistent with transformation efficiency (Fig. 8B). However, the difference in transformation efficiency of wild-type strain between glucose and galactose conditions was significantly higher than *ccpA*-deficient strain, indicating that carbohydrates did affect the regulation of *ccpA* on transformation. Interestingly, bacterial transformation efficiency and *comCDE* transcription decreased with increasing carbohydrate concentrations (glucose/galactose) (Fig. 8C and D). The inhibitory effect of galactose on transformation and *comCDE* transcription could be attributed to the reduction of energy supply. Because energy supply is required for pneumococci to enter competence and for the uptake of transforming DNA, most of the ATP supply in S. pneumoniae is derived from the glycolytic breakdown of glucose (62). However, it is not clear why the *comCDE* transcription and transformation decrease with increasing glucose concentration.

Therefore, we further explored how glucose affects CcpA-mediated regulation. HPr is another component of the PTS and can regulate the activity of CcpA bound to cre. In the presence of preferred carbohydrate, HPr can be phosphorylated at Ser-46 (P-Ser-HPr), which then allows binding to CcpA (59, 60). The complex of CcpA and P-Ser-HPr bind to cre sites on the DNA and thereby represses the transcription of catabolic genes (carbon catabolite repression [CCR] effects) (60, 63). Therefore, glucose may affect CcpA-mediated regulation by affecting the phosphorylation of HPr at Ser-46. We introduced mutations in HPr at the regulatory serine residue that is classically used to investigate HPr-mediated CCR in Gram-positive species and generated the mutants HPr S46A and HPr S46D (64, 65). HPr S46A cannot be phosphorylated and hence cannot exclude inducers or corepress with CcpA. The mutant HPr S46D served as a phosphomimetic mutation giving the opposite phenotype: perpetual exclusion of inducers and corepression with CcpA (66). We analyzed the effect of the HPr S46D mutant on transformation and *comCDE* transcription because the HPr S46A mutation was lethal. The transformation efficiency (Fig. 8E) and *comCDE* transcription (Fig. 8F) of strain HPr S46D strain was significantly lower than wild type. When we overexpressed CcpA in the HPr S46D mutant, transformation (Fig. 8E) and *comCDE* transcription (Fig. 8F) were both increased compared with HPr S46D mutant. This indicated that CcpA could partially reverse the inhibitory effect of P-Ser-HPr. Taken together, the above results indicated that glucose can via HPr S46D alter CcpA-mediated regulation to P*comCDE*, thus affecting *comCDE* transcription and transformation.

## DISCUSSION

**FIG 9:**
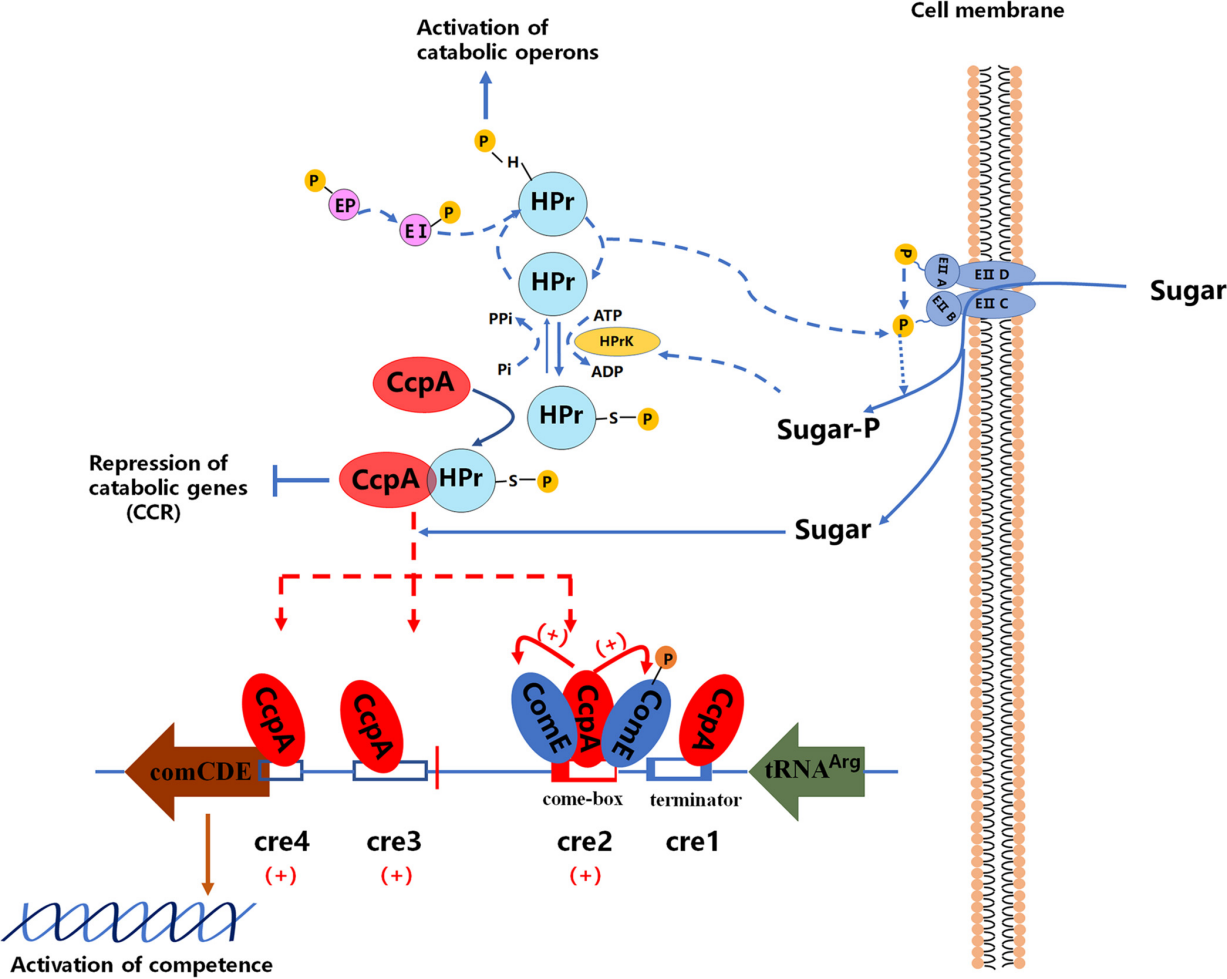
A working model of CcpA-regulated competence in S. pneumoniae D39. The *comCDE* operon is the core regulatory element of competence. S. pneumoniae controls competence development through basal and autoregulatory transcription of the *comCDE*. There are four CcpA-bound cre sites on P*comCDE*, and except for cre1, CcpA participates in *comCDE* basal and autoregulatory transcription via binding to by binding to these sites. P*comCDE* initiates the autoregulated transcription that is enhanced by phosphorylated ComE but inhibited by dephosphorylated ComE. CcpA can cooperate with phosphorylated and dephosphorylated ComE to bind P*comCDE* to maintain the optimal *comCDE* transcription. Remarkably, acting as a global regulator of carbohydrate metabolism genes, CcpA can regulate *comCDE* transcription by responding to the availability of carbohydrates. PTS delivers the phosphoryl group provided by PEP to HPr, enzyme I (EI), and enzyme II (EII) in turn and mediates carbohydrate transport across the cell membrane. In the presence of preferred carbohydrate, HPr can be phosphorylated at Ser-46, and P-Ser-HPr binds to the CcpA protein. The complex of CcpA and P-Ser-HPr binds to cre sites on the DNA and thereby represses the transcription of catabolic genes (CCR effects). This process can affect the binding of CcpA to cre in P*comCDE*, thus regulating the *comCDE* transcription. However, in the absence of preferred carbohydrate, HPr can be phosphorylated at His-15, and P-His-HPr phosphorylates contribute to the activation of catabolic operons. Activation of the nonpreferred carbohydrate metabolism can inhibit the transcription of *comCDE*. Finally, CcpA affects competence by regulating *comCDE* transcription. CCR, carbohydrate catabolite repression.Cre, catabolite response elements.

S. pneumoniae is a major human respiratory pathogen that causes serious infections with high levels of mortality and morbidity (15). Its success as a pathogen lies in its ability to metabolically adapt to diverse infection niches and acquire antibiotic resistance via HGT (17, 18). Natural competence is crucial in promoting HGT, and the *comCDE* operon is the core regulator of competence in S. pneumoniae. Only ComE has been shown to directly regulate *comCDE* transcription. CcpA is a member of the LacI/GalR family of transcriptional regulators that can act as either a positive or a negative regulator of genes that are in most cases involved in carbohydrate acquisition or metabolism (63). In this study, we show a link between CcpA and competence (Fig. 9).

Natural transformation was first found in S. pneumoniae in 1928, and at least 83 bacterial species have been found capable of natural transformation (67). Although similar mechanisms are employed to acquire and recombine genetic material into their genomes, only a few bacteria have the ability to transform naturally throughout their growth period, such as Neisseria gonorrhoeae (68) and Acinetobacter baumannii (69). The remainders are capable of natural transformation only at particular growth stages, and S. pneumoniae undergoes natural transformation during early exponential growth (27). Competence is a prerequisite of natural transformation. The induction of competence in S. pneumoniae is divided into two temporally distinct phases, early and late, regulated by ComE~P or ComX, respectively (27, 70). Peterson confirmed in TIGR4 through DNA microarray that the transcription of early and late competence genes peaked at 9 and 12 min after CSP induction, respectively. After 40 min, their expression rapidly decreases, and competence is shut down (27). Our results demonstrated a similar pattern of competence development in S. pneumoniae D39. The early competence genes (*comE*, *comW*, and *comX1*) and late competence genes (*recA*, *ssbB*, and *dprA*) were rapidly induced, reaching peak levels at 5 and 10 min, respectively, followed by a rapid decay at 30 min. The *ccpA* deletion altered the dynamic development pattern of competence; the transcription level of late competence genes, especially, was reduced. Late competence genes are mainly involved in DNA uptake and internalization, so the reduced transformation efficiency of *ccpA*-deficient bacteria can be attributed to the reduced transcription of late competence genes. However, the mRNA expression of early competence genes (*comE*, *comW*, and *comX1*) in Δ*ccpA* slightly decreased compared to the WT. This might be attributed to the fact that *comX* is a highly efficient transcription factor, and minor alterations also lead to amplification of downstream signaling cascades, thus making late competence genes significantly different. In the study of Martin et al. (22), the transcription of early genes (P*comX* and P*comW*) was slightly altered between strains tested, but the transcription of late genes (P*ssbB*) changed significantly. This result was consistent with the above hypothesis.

At the same time, we found that the *comE* mRNA of the wild-type strain decreased rapidly after 10 min of CSP induction, and the *comE* mRNA degradation of the *ccpA* deletion strain slowed down, suggesting that there may be a post-transcriptional regulation mechanism for the degradation of *comE* mRNA, and CcpA may positively regulate this post-transcriptional regulation. Thus, in the wild-type strain, increased *comCDE* transcription was followed by increased *comE* mRNA levels, but unknown post-transcriptional regulation reduced *comE* mRNA by rapid degradation. In the *ccpA*-deficient strain, low *comCDE* transcriptional activity leads to slow increase of *comE* mRNA, and the inhibition of *comE* post-transcriptional regulation leads to slow degradation of *comE* mRNA. This may also be one of the reasons why CcpA has the optimal concentration effect on the competence regulation. When the concentration of CcpA is too high, the degradation of *comE* mRNA will increase, so that the ComE protein will not increase and may even decrease, and ultimately the transformation rate will not increase. At present, only five cia-dependent small RNAs (csRNAs) regulated by *ciaRH* TCS have been reported to regulate the *comCDE* operon via post-transcription. They block CSP precursor production by binding around the *comC* translation initiation region without degrading *comC* mRNA (71, 72). Our RNA-seq showed no difference in *ciaRH* expression between wild-type and *ccpA*-deficient bacteria. Therefore, there may be an unknown factor regulating the degradation of *comE* mRNA, which is worthy of further study.

CcpA employed two different binding motifs (one is a typical cre and the other one is an atypical cre) to activate or repress target gene transcription (57, 73). We confirmed that CcpA is involved in the basal and autoregulatory transcription of the *comCDE* by binding to these atypical cre in *PcomCDE*, respectively. cre1 is located upstream of the P*comCDE* in the tRNA^Arg^ gene TRT region. In S. pneumoniae, TRT of the upstream tRNA^Arg^ gene has been reported to regulate one-third of the *comCDE* basal transcription (19). In Streptococcus oligofermentans, CcpA controls the basal *comCDE* transcription by regulating tRNA^Arg^ TRT (34). In this study, CcpA binding to cre1 did not initiate *comCDE* transcription. It is therefore not surprising that no transformants or ComE are observed. cre2 is located in the ComE box region, and CcpA increased the *ssbB* transcription in a concentration-dependent manner after binding to cre2, suggesting that this binding increased phosphorylated ComE expression. CcpA can also promote the *comCDE* transcription by binding cre3 and cre4, showing an optimal concentration effect. The transformation and *ssbB* transcription after CcpA binding to cre4 were significantly higher than other sites, indicating that CcpA may regulate the competence mainly through binding to cre4. However, when the IPTG concentration was at 50 μM, the transformation and *ssbB* transcription did not increase after CcpA binding to cre4, which may be one of the reasons for the optimal concentration effect of CcpA on competence regulation.

There are two forms of ComE: phosphorylated and unphosphorylated. Phosphorylated ComE (ComE~P) specifically induces the expression of 25 genes, including the comAB and *comCDE* operons, which generates a positive feedback loop that controls competence development. Conversely, unphosphorylated ComE acts as repressor of its own regulon. The development of competence is modulated by the ComE/ComE~P ratio (22). We demonstrated that CcpA enhances *comCDE* transcriptional of phosphorylated ComE by constructing ComE^D58E^ mutants, and CcpA can also enhance the inhibitory effect of ComE^D58A^ on *comCDE* transcription. Either too low or too high expression of CcpA will lead to the imbalance of comE/comE~P ratio, affecting the *comCDE* transcription and subsequently affecting the transformation. This may also be one of the reasons for the optimal concentration effect of CcpA.

In some Gram-negative species, carbohydrate catabolite repression (CCR) is involved in the regulatory network governing natural competence and transformation (33, 74–76). The CCR mechanism is widely employed by bacteria to repress the utilization of nonpreferred carbohydrates such as galactose and mannose (77). The CcpA function as the central regulators of CCR in S. pneumoniae. However, it remains unknown whether CCR regulates competence and transformation in S. pneumoniae. In the present study, RNA-seq revealed that competence genes involved in DNA uptake and recombination were downregulated after *ccpA* deficiency, consistent with its reduced transformation efficiency. Interestingly, the expression of genes encoding nonpreferred carbohydrate transport-related genes were significantly increased, suggesting that the CCR effects on nonpreferred carbohydrates were suppressed after *ccpA* deletion. The transformation efficiency and *comCDE* transcription of S. pneumoniae under glucose conditions were significantly higher than those under galactose conditions. These results all support the link between CCR and competence in S. pneumoniae. A variety of carbon sugars are available in the host niche. The present study focused only on the effects of glucose and galactose on the CcpA-comCDE axis. More experiments are needed to investigate the regulatory effects of other carbon sources on the CcpA-comCDE axis.

The CCR control of carbohydrate metabolism by CcpA also requires the participation of the HPr protein. HPr has two potential sites of phosphorylation: His-15 and Ser-46. When a preferred carbohydrate such as glucose exists, high levels of intracellular ATP/P_i_ and glucose metabolites activate the kinase activity of HPrK/P to phosphorylate the serine residue and then allows binding to CcpA to form a complex and participates in the CCR effect (78, 79). CCR is relieved when decreased availability of the preferred carbohydrate results in decreased flux through glycolysis and hence less stimulation of HPrK/P kinase activity. Under these circumstances, HPrK/P resorts to its phosphorylase activity and dephosphorylates HPr-Ser~P, replenishing the supply of HPr phosphorylated on His-15 to participate in PTS transport (nonpreferred carbohydrates) (66). Transport of nonpreferred carbohydrates resumes, and CcpA-dependent CCR effect is relieved. Our results demonstrated that the transformation efficiency and *comCDE* transcription of P-Ser-HPr strains were inhibited. This explains why transformation efficiency and *comCDE* transcription of S. pneumoniae was inhibited by increased glucose levels, because glucose metabolites promote the generation of P-Ser-HPr (78, 79). However, the reason P-Ser-HPr inhibits transformation is not clear. Interestingly, overexpression of *ccpA* in the P-Ser-HPr strain (P-Ser-HPr-CcpA) increased both *comCDE* transcription and transformation efficiency. These results indicate that part of P-Ser-HPr binds to CcpA to form a complex and participates in the CCR effect that can offset P-Ser-HPr inhibition of transformation, thus increasing the *comCDE* transcription and transformation efficiency. Energy supply is also required for pneumococci to enter competence and for the uptake of transforming DNA (62). Most of the ATP supply in S. pneumoniae is derived from the glycolytic breakdown of glucose. Therefore, CcpA may also increase energy production by mediating the CCR effect and may contribute to the development of competence, but this needs to be confirmed by further experiments.

Under the current experimental evidence, we find that CcpA as regulator of pneumococcal competence is for the particular case of D39. In future studies, we will further investigate the regulatory role of CcpA on *comCDE* transcription in other serotypes. CcpA acts as a global regulator of carbohydrate metabolism genes and regulates dozens of metabolism and virulence genes (36–38). In this work, we revealed that CcpA was involved in the regulation of competence of S. pneumoniae D39, which broadens our understanding of the function of CcpA and provides valuable experimental evidence for subsequent studies on bacterial resistance, evolution, and pathogenicity.

## MATERIALS AND METHODS

### Strains and culture conditions.

The bacterial strains and plasmids used in this study are listed in Table S1. All S. pneumoniae strains were grown at 37°C under 5% CO_2_ in C+Y medium or plated on blood agar plates (Autobio Biotech, Zhengzhou, China). Frozen bacterial stocks were prepared by growing the strains in C+Y to an OD_600_ of 0.5 before addition of glycerol to a final concentration of 15% and storing at −80°C. Escherichia coli strains used for cloning and plasmid amplification were grown in Luria-Bertani broth or agar plates at 37°C. When appropriate, antibiotics were added to the growth medium, as shown in Table S1.

### Transformation efficiency assay.

Pneumococcal stocks were inoculated in C+Y medium. To maintain the same density at the time of competence induction, the cells were grown to an OD_600_ of 0.1, and 100 μL was harvested. CSP1 (EMRLSKFFRDFILQRKK) was purchased from Sangon Biotech (Shanghai, China) and used at 10 μg/mL. Subsequently, the cells were incubated 10 min at 37°C before the addition of 200 ng transforming DNA and then incubated 2 h at 37°C. For transforming DNA, we used gDNA (containing erythromycin resistance) and plasmids (pPEPZ-plac and PJWV25). After incubation for an additional 2 h at 37°C, the cells were vortexed, and 10 μL aliquots of each sample was 10-fold serially diluted followed by plating on nonselective blood agar plates for counting of the total bacterial numbers. The remaining sample was plated on antibiotics containing blood agar plates, and positive transformants were counted. Transformation efficiency was calculated as the number of antibiotic-resistant CFU relative to the number of CFU on nonselective blood agar plates.

### Construction of mutants.

S. pneumoniae strains are listed in Table S1. Mutants were generated by homologous recombination using the primers listed in Table S2. All mutant strains originate from the D39s strain, a streptomycin-resistant derivative of D39 referred to as the WT. The Δ*ccpA* strain was generated in a two-step transformation procedure. The upstream and downstream homologous arms of the *ccpA* locus were amplified from D39 genomic DNA with primer pairs *ccpA*-UP F/Pr1328 and Pr1329/*ccpA*-down R, respectively. The Janus cassette was amplified with primers Pr1332 and Pr1333 from genomic DNA of strain ST588 (80). The Janus cassette, which has kanamycin resistance and a dominant *rpsL* allele, was utilized for selection of kanamycin-resistant, streptomycin-sensitive colonies. In contrast, unmarked strains are kanamycin-sensitive and streptomycin-resistant. Fusion PCR was performed with the upstream arm, the Janus cassette, and the downstream arm with primer pairs *ccpA*-UP F and *ccpA*-down R and transformed into strain D39s to construct Δ*ccpA*::kan-rpsL (Δ*ccpA*::JC) (39). To generate unmarked deletions in the *ccpA locus*, the up- and downstream sequences were amplified with the primer pairs *ccpA*-UP F/Pr1330 and Pr1331/*ccpA*-down R. The up- and downstream amplicons were ligated by fusion PCR with primers *ccpA*-UP F and *ccpA*-down R and transformed into strain D39s to construct *ccpA* unmarked deletion strain (Δ*ccpA*).

The *ccpA* complemented strain was constructed using a DNA fragment containing the promoter and coding region of the *ccpA* gene that was PCR amplified. The purified PCR fragment was double digested with BamHI and XhoI and then inserted into the shuttle plasmid pIB166 that had been digested with the same enzymes to produce pIB166-*ccpA*. Recombinants were confirmed by restriction analysis, PCR, and sequencing. The recombinant plasmids pIB166-*ccpA* was transformed into the Δ*ccpA* mutant strain to make the *ccpA* complemented strain (Δ*ccpA*::*ccpA*).

Site-directed mutagenesis of comE involved the use of the PCR-based gene splicing by overlap extension method. Briefly, a couple of PCRs (with primer pairs A1-A2 and B2-B1) were used to generate two fragments A and B that incorporated a mutant primer (A2) at one extremity of A and its complement (B2) at the other extremity of B. A third PCR with primer pair A1–B1 then produces a unique fragment with the mutant sequence in the middle. Primer pairs Pr1429/comE^D58E^-m2 and comE^D58E^-m3/Pr1430 generate the comE^D58E^ fragment. Primer pairs Pr1429/Pr1426 and Pr1425/Pr1430 generate the comE^D58A^ fragment. To construct a comE^D58E^ strain, the comE^D58E^ PCR fragment was digested with BglII and XhoI generating a 787-bp fragment, which was ligated to BglII-XhoI-digested pPEPZ-plac plasmid DNA to construct plasmid pPEPZ-comE^D58E^. The *lacI* gene was amplified with primers Pr1951 and Pr1952 from genomic DNA of plasmid pPEPY-PF6-*lacI* and transferred into the wild-type and Δ*ccpA* strains to construct D39s-lacI and D39Δ*ccpA*-*lacI*, respectively. Subsequently, the recombinant plasmid pPEPZ-*comE*^D58E^ was transformed into the D39s-*lacI* and D39Δ*ccpA*-*lacI* mutant strains, with the expression of *comE*^D58E^ under IPTG control. The construction method of the comE^D58A^ mutant was the same as *comE*^D58E^ mutant.

We constructed IPTG-inducible strains of *ccpA* to generate the CEPlac expression platform. A DNA fragment containing Ps-ami-lacI and Plac with primer pairs Pr1371/Pr1375 was synthesized. This fragment was digested with ApaI and BamHI and ligated into the shuttle plasmid pIB166-*ccpA* digested with the same enzymes to form the plasmid pIB166-CEPlac-*ccpA*. This plasmid pIB166-CEPlac-*ccpA* was transformed into D39Δ*ccpA*-*lacI* mutant to generate the Δ*ccpA*::CEPlac-*ccpA* strain with *ccpA* expression under IPTG control.

### Construction of luciferase reporter strains and luciferase assay.

Luciferase reporter constructs of the *comCDE* promoter region were prepared in the integrative plasmid pEVP3 (81). The firefly luciferase gene (*luc*) was amplified from plasmid pR424 (82) and cloned into a 2,152-bp-long BamHI-NotI fragment from plasmid pEVP3 to generate plasmid pEVP3-luc. The *comCDE* promoter sequence was amplified from genomic DNA of strain D39 using primer pairs Pr1311/Pr1312 and digested with XhoI-BamHI to generate a 410-bp fragment. This fragment was cloned into a 3,738-bp-long XhoI-BamHI fragment from plasmid pEVP3-luc and confers chloramphenicol resistance to generate plasmid pEVP3-Pcom-luc. A ssbB-luc transcriptional fusion was generated in plasmid pEVP3-luc in a similar fashion. A DNA fragment overlapping the 5′ end of the *ssbB* gene was amplified from D39 chromosomal DNA by PCR using primer pairs Pr1401/Pr1402. The *ssbB* sequences were digested with XhoI/BamHI and ligated to XhoI/BamHI-digested pEVP3-luc, resulting in plasmid pEVP3-PssbB-luc. The luciferase assays were conducted in the presence of 0.66 mM d-luciferin (Beyotime, Shanghai, China) solution as described previously (82). The optical density (OD_600_) of the samples was measured and used to normalize the luciferase activity.

### Recombinant protein production.

The pneumococcal *ccpA* gene from S. pneumoniae D39 was amplified by PCR, sequenced to ensure accuracy, and then cloned into the pET-28a expression vector and into E. coli BL21(DE3). The expression of CcpA recombinant protein was induced in E. coli BL21 grown in 1 liter Luria-Bertani medium supplemented with 50 μg/mL kanamycin and 1 mM isopropyl-β-d-thiogalactopyranoside (IPTG). The CcpA protein was collected and purified by affinity chromatography with an Ni^2+^/nitrilotriacetic acid (NTA) column. ComE and ComE^D58E^ proteins were expressed in the same way.

### Quantification of capsule.

Analysis of pneumococcal CPS using uronic acid assay. CPS samples were prepared by resuspending pneumococci grown in C+Y medium to an OD_600_ of 0.1. An aliquot of 5 mL was pelleted in a bench centrifuge at 3,000 × *g* for 20 min and resuspended in 500 μL of 150 mM Tris·HCl (pH 7.0) and 1 mM MgSO_4_. The samples were then treated with 0.1% (wt/vol) deoxycholate (Sigma), 100 units of mutanolysin (Sigma), 50 μg DNase I (Roche Applied Science), 50 μg RNaseA (Roche Diagnostics), and 50 μg proteinase K (Roche Diagnostics). The amount of CPS present in each sample was determined by using an assay for quantitative determination of uronic acids as described by Blumenkrantz and Asboe-Hansen (83).

Analysis of pneumococcal CPS using enzyme-linked immunosorbent assay (ELISA). S. pneumoniae was incubated in C+Y medium to an OD_600_ of 0.1. After centrifugation at 4,000 × *g* for 10 min, the bacterial pellets were washed three times with PBS. The pellets from 1 mL of culture were suspended in 500 μL of PBS, and the optical density was adjusted to obtain similar amounts of bacteria. To measure total CPS, an indirect ELISA was performed as previously described (84).

### Electrophoretic mobility shift assays (EMSAs).

The promoter regions of the *comCDE* operons were PCR amplified from S. pneumoniae D39 using primers Bio-comCDE F/Bio-comCDE R (Table S2). The primers were biotin labeled by the Tsingke Biotechnology (Beijing, China). Labeled probes were incubated with increasing concentrations of purified CcpA in binding buffer (10 mM Tris-HCl, pH 7.4, 1 mM dithiothreitol, 1 mM EDTA, 50 mM KCl, 5% glycerol, 50 μg/mL bovine serum albumin, 0.05% Nonidet P-40) for 20 min at 37°C as described for the protein-DNA binding interaction assay kit (Thermo Fisher). After incubation, the samples in bromophenol blue loading buffer were separated by electrophoresis using 5% acrylamide gels in 0.5× Tris-borate-EDTA buffer that had been prerun in 0.5× TBE. The samples were electroblotted from the gels onto nylon membranes (Bio-Rad) and UV cross-linked, and the bands were visualized with a chemiluminescence substrate and captured using the Chemiluminescence imaging system (Bio-Rad).

### DNase I footprinting assay.

DNase I footprinting assays were performed at Tolobio (Anhui, China). Briefly, 300 ng probes were incubated with different amounts of protein in a total volume of 40 μL and incubated for 30 min at 25°C and 10 μL solution containing 0.015 U DNase I (Promega, Madison, WI, USA) and 100 nmol freshly prepared CaCl_2_ was added, and further incubation was performed at 37°C for 1 min. The samples were extracted with phenol-chloroform and precipitated with ethanol, and the pellets were dissolved in 10 μL Mini-Q water. The preparation of the DNA ladder, electrophoresis, and data analysis were performed as previously described (85) except the GeneScan-LIZ600 size standard (Applied Biosystems) was used.

### Western blotting.

S. pneumoniae was grown in C+Y medium at 37°C to an OD_600_ of 0.1 and then treated with CSP for 10 min. The samples were centrifuged, and the cell pellets were lysed with lysis buffer (0.5% deoxycholate). The protein concentration was measured using the NanoDrop spectrophotometer, and the samples were subjected to SDS-PAGE and electrotransferred onto 0.2 μm polyvinylidene difluoride (PVDF) membranes (Merck Millipore, Burlington, MA, USA). To detect CcpA and ComE, purified CcpA and ComE were used to immunize C57BL/6 mice to generate polyclonal antibodies that were used diluted at 1:1,000. The proteins were visualized by adding Immobilon Western horseradish peroxidase (HRP) substrate peroxide solution (Millipore) captured using Image Lab software (Bio-Rad).

### Coimmunoprecipitation.

For coimmunoprecipitation, cultures of S. pneumoniae cells were grown at 37°C in C+Y medium containing 1 mM IPTG to OD_600_ 0.1. The cells were collected by centrifugation (8,000 × *g* for 10 min at 4°C). The cell pellets were washed once with 30 mL of 1× PBS (4°C) and resuspended in 2 mL of cold lysis buffer (Beyotime, P0013) with protease inhibitor (Beyotime, P1065). Cell debris and lysing matrix from tubes were removed by centrifugation at 14,000 × *g* for 15 min at 4°C. The protein concentration of each sample was determined by ThermoFisher protein assay (ThermoFisher Scientific), and 1 mL of lysate with equal amounts of total protein (5 mg/mL) was then incubated with protein G-agarose (Millipore, 2465262) coupled with 20 μg anti-His antibodies (Sangon Biotech, D191001) and incubated for 2 h at 4°C. After extensive wash with ice-cold cell lysis buffer, protein-bounded beads were eluted with SDS-PAGE loading buffer at 95°C for 5 min and analyzed by SDS-PAGE.

### RNA extraction and qRT-PCR.

Total RNA was extracted from S. pneumoniae cultures harvested in the early log phase (OD_600_ = ~0.10) using RNAprep Pure Cell/bacteria kit (Tiangen, Beijing, China) according to the manufacturer’s instructions. The RNA concentration was measured using a NanoDrop spectrophotometer (Thermo Fisher, Pittsburg, PA, USA), and its integrity was confirmed by agarose gel electrophoresis. RNA was converted to cDNA using PrimeScript RT reagent kit (TaKaRa, Beijing, China). Quantitative reverse transcription-PCR (qRT-PCR) was run and analyzed using CFX Maestro software (Bio-Rad, Hercules, CA, USA) using SYBR green qPCR Master Mix (Bimake, Houston, TX, USA) according to the manufacturer’s instructions using gene-specific primers (Table S2). Relative amplification was calculated using the 2^–ΔΔCT^ Livak method, and the gyrB rRNA gene was used as an internal reference.

### RNA-seq.

S. pneumoniae D39 (WT) and Δ*ccpA* strains were grown in normal C+Y culture to an OD_600_ of 0.1, and their total RNA was extracted after 10 min of CSP treatment. Total RNA was extracted using RNAprep Pure Cell/bacteria kit (Tiangen) according to the manufacturer’s instructions. All extracts were submitted to the Novogene Corporation (Beijing, China) for RNA-seq analysis. Trimmed reads were mapped to the genome of S. pneumoniae D39 as the reference genome (GenBank accession number NC_008533.2). RNA-seq data were deposited as BioProject no. PRJNA929078.

### Statistical analysis.

Unless otherwise specified, all remaining data are expressed as means ± SD and analyzed by two-tailed Student’s *t* test. *P* values of <0.05 were considered statistically significant.
