# Supplementary material for: comCDE (Competence) Operon Is Regulated by CcpA in Streptococcus pneumoniae D39
Source: Microbiol Spectr. 2023 Apr 10;11(3):e00012-23. doi: 10.1128/spectrum.00012-23 (PMC10269683; doi:10.1128/spectrum.00012-23)
Supplement: Supplemental file 1 — Supplemental material. Download spectrum.00012-23-s0001.pdf, PDF file, 0.9 MB [file spectrum.00012-23-s0001.pdf]

Supplemental Material

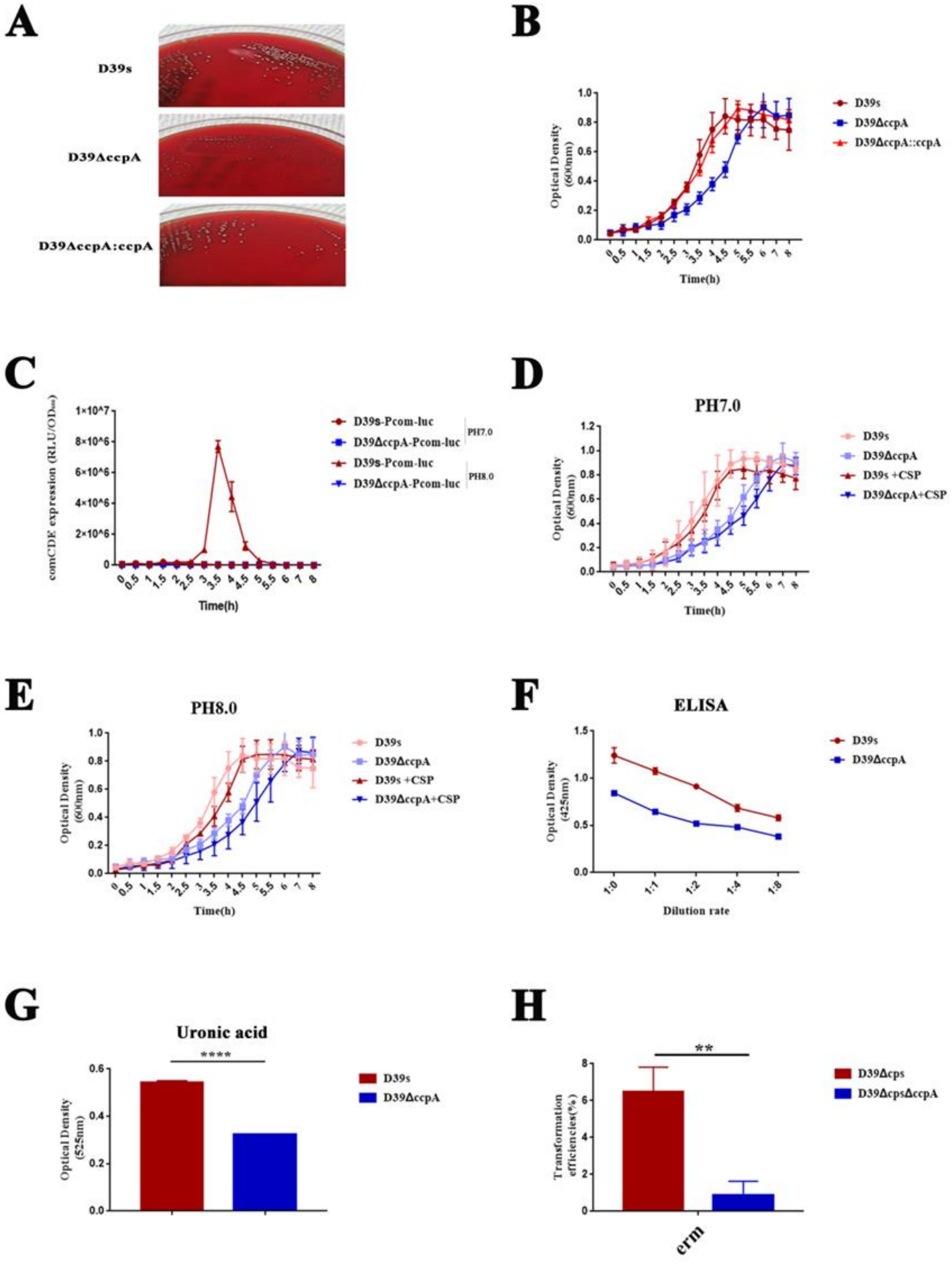

Figure S1. Links between growth phenotype and competence. (A) Colony morphology on a blood agar plate of D39s, D39*ΔccpA* and D39ΔccpA::ccpA strains cultured overnight. **(B)** Growth curves of wild-type and *ccpA* mutants in C+Y medium. **(C)** Transcription profiles of the *comCDE*

operon in wild-type strain and *ccpA* deletion mutant during growth in C+Y medium at permissive pH (pH 8.0) and non-permissive pH (pH 7.0) were determined using luciferase reporter strains D39s-Pcom-luc and D39Δ*ccpA*-Pcom-luc, which were constructed by fusing the luciferase gene with the *comCDE* promoter. *comCDE* transcription was reported by luciferase activity (relative light units, RLU per OD_{600}) of D39s-Pcom-luc and D39Δ*ccpA*-Pcom-luc strains respectively. **(D and E)** Growth curves of wild-type and *ccpA* mutants in C+Y medium at pH 7.0 (D) and 8.0 (E). Overnight cultures of the strains were each diluted into fresh C+Y medium and cultured at 37°C. After culturing for 1 h at 37 °C, 200 ng of CSP was added and the OD_{600} was recorded every 30 min for a total of 8 h. No CSP treatment group was used as a control. **(F and G)** Capsule was measured with ELISA (F) and uronic acids methods (G). Cells were collected when they were grown in C+Y medium to an OD_{600} of 0.1. **(H)** Effect of *ccpA* on the transformation of unencapsulated *S. pneumoniae* using gDNA as DNA donors. *, $p<0.05$; **, $p < 0.01$; ***, $p < 0.001$, ***, $p < 0.0001$.

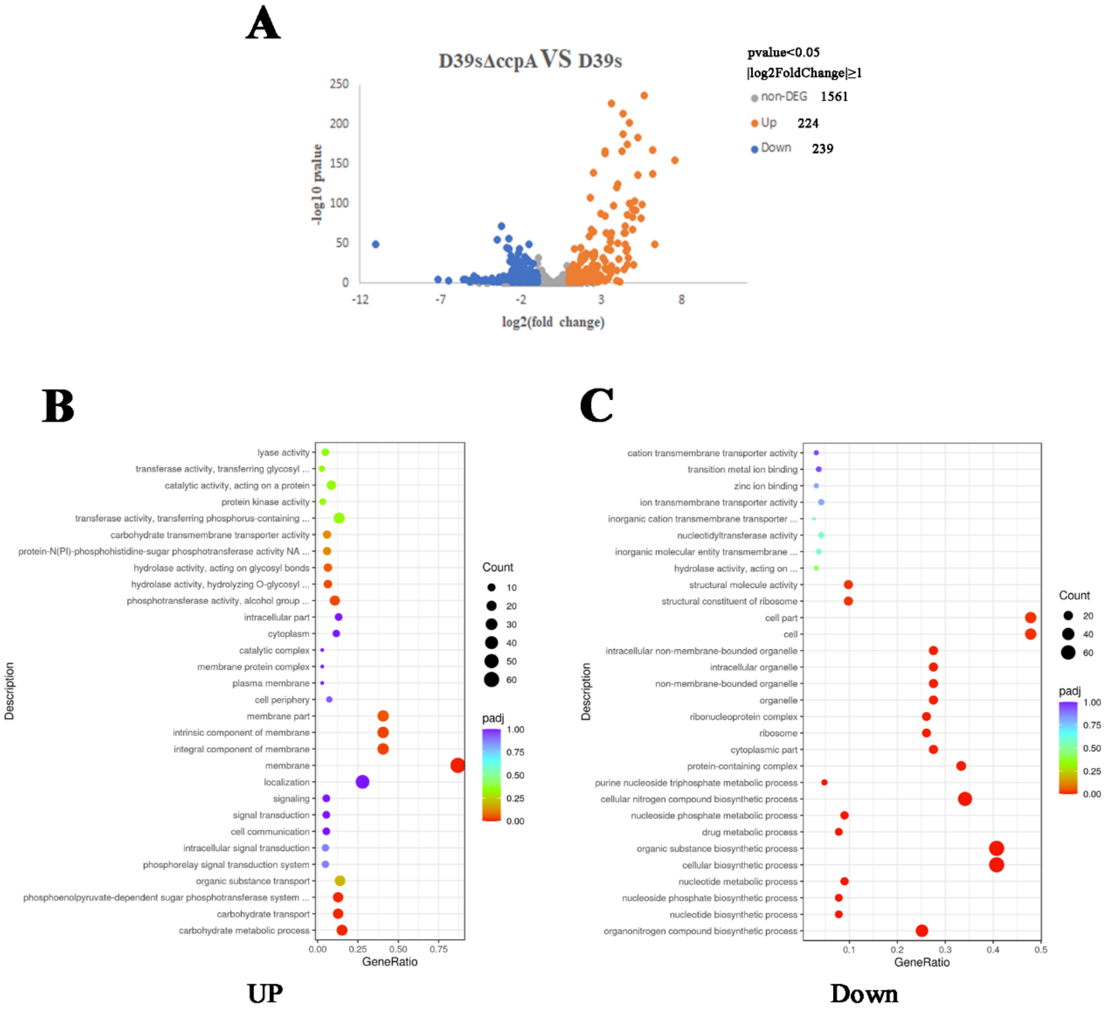

Figure S2. Comparison of differentially expressed genes between D39s and D39ΔccpA by RNA-seq. **(A)** Volcano plot of differentially expressed genes. The abscissa represents the ratio of differentially expressed genes in CSP-treated D39*ΔccpA* versus CSP-treated D39s; the ordinate represents the P value between the two groups. **(B)** GO enrichment cluster analysis of up-regulated genes. **(C)** GO enrichment cluster analysis of down-regulated genes.

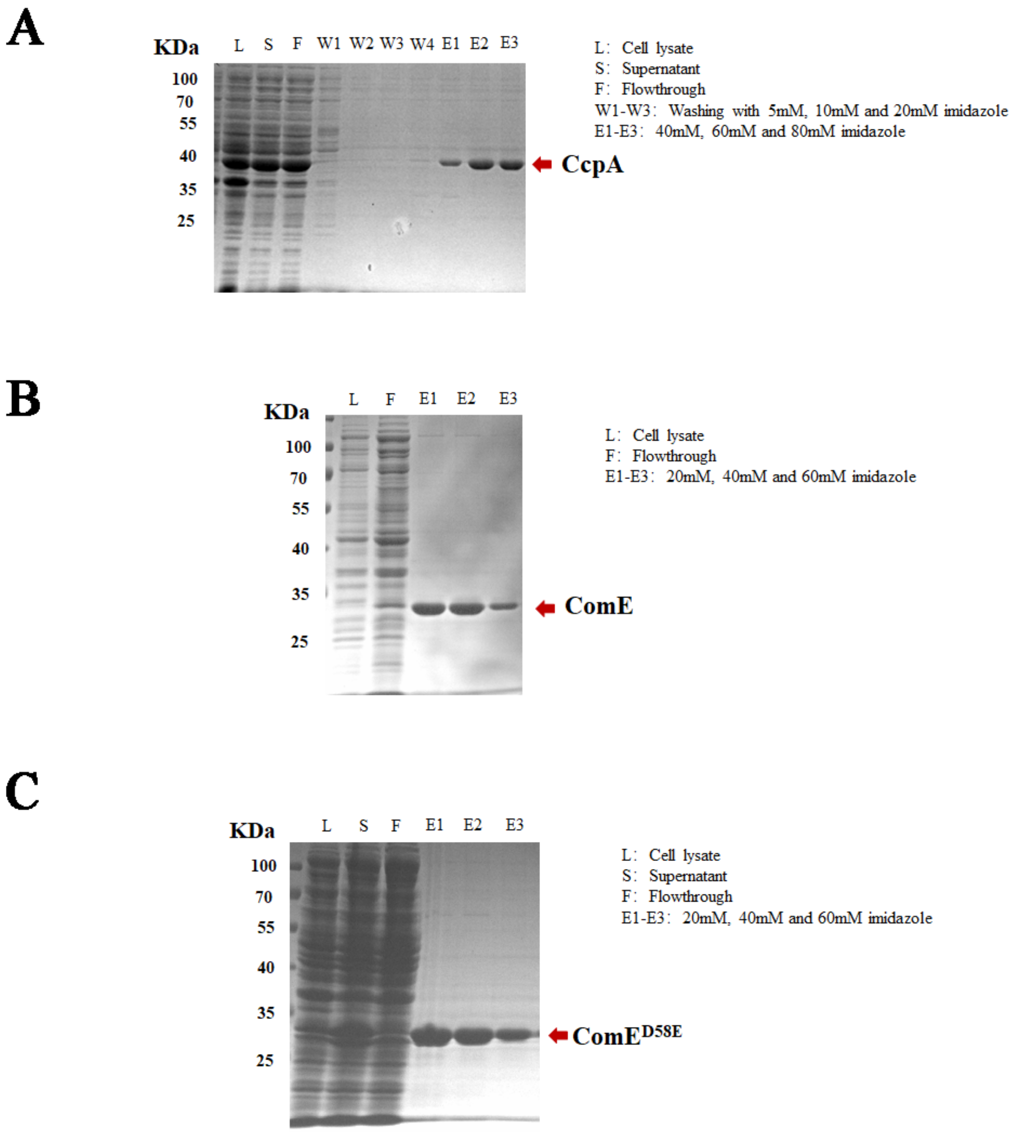

Figure S3. Overproduction and purification of CcpA, ComE and ComE^{D58E}. SDS-PAGE analysis of **(A)** CcpA **(B)** ComE and **(C)** ComE^{D58E} at the indicated purification steps. The CcpA, ComE or ComE^{D58E} are marked with red arrowheads. A 6×His-tagged version of CcpA, ComE and ComE^{D58E} was overproduced in *E. coli* and purified using a Ni-binding resin.

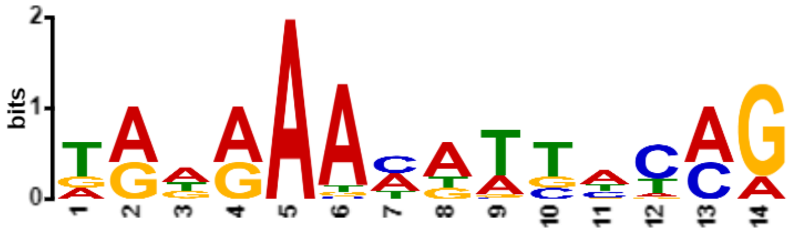

**typical cre**	TGWAANCGNTNWCA
**Cre 1**	GCAAAGCTGGGAATTTTCCCGGCTTTTTTCTTAAAA
**Cre 2**	TTTGGGAGAAAAAAATGACAGTTG
**Cre 3**	TAAGTTAGCTTACAAGAAAAAACATTTTAG
**Cre 4**	TATGAAAAACACAG

Figure S4. Alignment of cre sequences on P*comCDE* with the consensus CcpA-binding site. The MEME web server was used to conduct the alignment of cres sequences on P*comCDE* with typical CcpA-binding site. Typical cre is a typical consensus CcpA-binding site, the underlined nucleotides are involved in binding to CcpA, which N represents any base and W represents A or T. cre1, cre2, cre3, cre4 were atypical cres of CcpA binding to P*comCDE*.

Table S1 Bacterial strains and plasmids used in this study

Strain or plasmid	Description	Antibiotic resistance[a]	Reference or source
**Strains**			
D39	*S. pneumoniae* strain D39, serotype 2, encapsulated		NCTC
D39s	D39 derivative; rpsL1	Sm^R	[1]
D39ΔccpA::JC	D39s derivative; the *ccpA* region is replaced with Janus cassette	Kan^R	This study
D39ΔccpA	D39ΔccpA ::JC derivative; the entire *ccpA* region is removed	Sm^R	This study
D39ΔccpA::ccpA	D39ΔccpA ::pIB166-ccpA; D39ΔccpA derivative, *ccpA* ectopic expression	Cm^R	This study
D39ΔdexB-cps2A ::JC	D39s derivative; the dexB-cps2A region is	Kan^R	This study

	replaced with JC		
D39Δcps	D39ΔdexB-cps2A ::JC derivative; the entire dexB-cps2A region is removed	Sm^R	[1]
D39ΔcpsΔccpA	D39Δcps derivative; the entire ccpA region is removed	Sm^R	This study
D39s-lacI	D39s derivative carrying *lacI* gene	$gent^R$	This study
D39ΔccpA-lacI	D39ΔccpA derivative carrying *lacI* gene	$gent^R$	This study
ΔccpA::CEPlac-*ccpA*	D39ΔccpA-lacI::pIB166-CEPlac-ccpA; D39ΔccpA-lacI derivative with the ccpA gene under the control of the lac promoter	Cm^R	This study
D39s-P_{com}-luc	D39s::pEVP3-Pcom-luc; D39s derivative with luc under the control of comCDE promoter	Cm^R	This study
D39ΔccpA-P_{com}-luc	D39ΔccpA::pEVP3-Pcom-luc; D39ΔccpA derivative with luc under the control of comCDE promoter	Cm^R	This study
D39s-P_{ssbB}-luc	D39s::pEVP3-P_{ssbB}-luc; D39s derivative with luc under the control of ssbB promoter	Cm^R	This study
D39ΔccpA-P_{ssbB}-luc	D39ΔccpA::pEVP3-P_{ssbB}-luc; D39ΔccpA derivative with luc under the control of ssbB promoter	Cm^R	This study
cre1	D39s-lacI:: pIB166-CEPlac-ccpA; only the cre1 region is retained in the comCDE promoter	Cm^R	This study
cre1(*)	D39s-lacI:: pIB166-CEPlac-ccpA; only the cre1 and transcription initiation region retained in the comCDE promoter	Cm^R	This study
cre2	*ΔccpA*::CEPlac-*ccpA* derivative; only the cre2 region is retained in the comCDE promoter	Cm^R	This study
cre3	*ΔccpA*::CEPlac-*ccpA* derivative; only the cre3 region is retained in the comCDE promoter	Cm^R	This study
cre4	*ΔccpA*::CEPlac-*ccpA* derivative; only the cre4 region is retained in the comCDE promoter	Cm^R	This study
D39-comE(His)	D39s-lacI derivative; with the comE-His gene under the control of the lac promoter and carrying lacI gene	$spec^R$	This study
D39-$comE^{D58E}$	D39s-Pcom-luc::pPEPZ-$comE^{D58E}$; D39s-Pcom-luc derivative with the $comE^{D58E}$ gene under the control of the lac promoter and carrying lacI gene	$spec^R$	This study
D39ΔccpA-$comE^{D58E}$	D39ΔccpA-Pcom-luc::pPEPZ-$comE^{D58E}$; D39ΔccpA-Pcom-luc derivative with the $comE^{D58E}$ gene under the control of the lac promoter and carrying lacI gene	$spec^R$	This study
D39ΔcomE::$comE^{D58E}$	D39-$comE^{D58E}$ derivative; the entire comE region is replaced by Janus cassette	Kan^R	This study
D39ΔccpAΔcomE::$comE^{D58E}$	D39ΔccpA-$comE^{D58E}$ derivative; the entire comE	Kan^R	This study

	region is replaced by Janus cassette		
D39-comE^{D58A}	D39s-Pcom-luc::pPEPZ-comE^{D58A}; D39s-Pcom-luc derivative with the comE^{D58A} gene under the control of the lac promoter and carrying lacI gene	spec^R	This study
D39ΔccpA-comE^{D58A}	D39ΔccpA-Pcom-luc::pPEPZ-comE^{D58A}; D39ΔccpA-Pcom-luc derivative with the comE^{D58A} gene under the control of the lac promoter and carrying lacI gene	spec^R	This study
D39ΔcomE::comE^{D58A}	D39-comE^{D58A} derivative; the entire comE region is replaced by Janus cassette	Kan^R	This study
D39ΔccpAΔcomE::comE^{D58A}	D39ΔccpA-comE^{D58A} derivative; the entire comE region is replaced by Janus cassette	Kan^R	This study
D39ΔHpr::JC	D39s derivative; the Hpr region is replaced with Janus cassette	Kan^R	This study
HPr S46D	D39ΔHpr::JC derivative; the Hpr region is replaced with Hpr serine residue phosphorylation gene	Sm^R	This study
HPr S46D-CcpA	HPr S46D::pIB166-ccpA; HPr S46D derivative and ccpA overexpression	Cm^R	This study
HPr S46D-P_{com}-luc	HPr S46D::pEVP3-Pcom-luc; HPr S46D derivative with luc under the control of comCDE promoter	Cm^R	This study
HPr S46D-CcpA-P_{com}-luc	HPr S46D-CcpA::pEVP3-Pcom-luc; HPr S46D-CcpA derivative with luc under the control of comCDE promoter	Cm^R	This study
**Plasmids**			
pIB166	*E. coli-S. pneumoniae* shuttle vector	Cm^R	[2]
pIB166-ccpA	*E. coli-S. pneumoniae* shuttle vector carrying the ccpA orf	Cm^R	This study
pR424	ColE1 derivative carrying an *S. pneumoniae* 5'-*ssbB*-targeting fragment driving *luc*	Cm^R	[3]
pEVP3	*E. coli-S. pneumoniae* integrative vector	Cm^R	[4]
pEVP3-luc	pEVP3 derivative carrying the luc reporter gene	Cm^R	This study
pEVP3-P_{com}-luc	pEVP3-luc derivative with the luc gene under the control of comCDE promoter	Cm^R	This study
pEVP3-P_{ssbB}-luc	pEVP3-luc derivative with the luc gene under the control of ssbB promoter	Cm^R	This study
pIB166-CEPlac-ccpA	pIB166 derivative with the ccpA gene under the control of lac promoter	Cm^R	This study
pPEPZ-Plac	Integrative plasmid with Plac promoter	spec^R	[5]
pPEPZ-comE^{D58E}	pPEPZ-Plac derivative carrying the comE^{D58E} orf	spec^R	This study
pPEPZ-comE^{D58A}	pPEPZ-Plac derivative carrying the comE^{D58A} orf	spec^R	This study
pPEPZ-comE(His)	pPEPZ-Plac derivative carrying the comE-his orf	spec^R	This study
pET-28a	protein expression vector	Kan^R	Takara

pET-28a-CcpA	pET-28a derivative carrying the CcpA orf fused to a (C-ter) His6 tag	Kan^R	This study
pET-28a-$comE^{D58E}$	pET-28a derivative carrying the $comE^{D58E}$ orf fused to a (C-ter) His6 tag	Kan^R	This study
pET-28a-ComE	pET-28a derivative carrying the ComE orf fused to a (C-ter) His6 tag	Kan^R	This study
pPEPY-PF6-lacI	This plasmid contains lacI gene driven by a strong constitutive promoter.	$gent^R$	Jingren Zhang laboratory
PJWV25	N-terminal GFP fusions under control of a Zn2+ inducible promoter, integrates via double crossover at bgaA	tet^R	[6]

Table S2 Primers used in this study

Primer		Sequence (5´-3´)
**Primer for Construction of series of mutant strains in D39**		
**JC**	Pr1332	TCTAGAGGATAATGCTGAAAACTCCTTGAAG
	Pr1333	CTCGAGCCTTTCCTTATGCTTTTGGAC
**ΔccpA**	Pr1328	CTTCAAGGAGTTTTCAGCATTATCCTCTAGATCCTTTTCCTGTCCTTTCTA
	Pr1329	GTCCAAAAGCATAAGGAAAGGCTCGAGAAAAAATCAGGGAATCGAGA
	Pr1330	TCTCGATTCCCTGATTTTTTTCCTTTTCCTGTCCTTTCTA
	Pr1331	ATAGAAAGGACAGGAAAAGGAAAAAAATCAGGGAATCGAGA
	ccpA-UP F	TTGTAGTGGCGTATTTGG
	ccpA-DW R	CAGCATTTCAAGGTCATTATC
**ΔccpA::ccpA**	ccpA-com F	CCGGAATTCATGAATGCAGATGATACAGTAACCA
	ccpA-com R	CCCAAGCTTCTATTTACGTTTTCGTGTTGAGCTA
**lacI**	Pr1951	GTGAAGTTATGAACATCATCGGTAAG
	Pr1952	GGTTCGCAAGCCATGGTTGGAG
ΔccpA*::CEPlac-*ccpA	Pr1371	GAGAGGGCCCGCTCTAGACGGTGATCAACACG
	Pr1372	ACTGTATCATCTGCATTCATAGATCCATTTGCCTCCTTAAAGATCT
	Pr1373	AGATCTTTAAGGAGGCAAATGGATCTATGAATGCAGATGATACAGT
	Pr1374	CGGGATCCGTGATGGTGATGGTGATGCTATTTACGTTTTCGTGTTG
	Pr1375	AGATACAAATCAAACAAATTTTGGGGTGATGGTGATGGTGATGCTATTTA
**pEVP3-luc**	Pr1301	TTGCGGCCGCCGCAGCCATGACCCAGTCAC
	Pr1302	GCTGGGGATCCATATGACGTCGACGCGTCT
	Pr1303	CGGGATCCAGGAGGAATAATGAGATCCG
	Pr1304	TTGCGGCCGCCTACGGGGATCTTACAATTT
**pEVP3-P_{com}-luc**	Pr1311	CCGCTCGAGCTAGTTCTTGTTGAACAAA
	Pr1312	AAGGCCTGGATCCTTCAAAGCTACAAACTGTTCC
**pEVP3-P_{ssbB}-luc**	Pr1401	CCGCTCGAGATTGGGCGTTTAACGTCTACACC
	Pr1402	CTCTCTGGATCCTCTTAAAATGGCAATTCTTCCTCTT
**cre1**	Pr1403	AAAGCTACAAACTGTTCCAATTTAAGTGTACTTTTTTAAGAAAAAAGCCG

	Pr1404	CGGCTTTTTCTTAAAAAAGTACACTTAAATTGGAACAGTTTGTAGCTTT
**cre1（*）**	Pr1413	AATTTCGTTTTAGATAAAATTCTCTGTGTACTTTTTTAAGAAAAAAGCCG
	Pr1414	CGGCTTTTTCTTAAAAAAGTACACAGAGAATTTTATCTAAAACGAAATT
**cre2**	Pr1405	AAAGCTACAAACTGTTCCAATTTAACAAAAACCATTATACAAAATGGAAT
	Pr1406	ATTCCATTTTGTATAATGGTTTTTGTTAAATTGGAACAGTTTGTAGCTTT
**cre3**	Pr1407	AATTTCGTTTTAGATAAAATTCTCTGTGTACTTTATTATATTGATCCCAG
	Pr1408	CTGGGATCAATATAATAAAGTACACAGAGAATTTTATCTAAAACGAAATT
**cre4**	Pr1409	AATTTCGTTTTAGATAAAATTCTCTGTGTACTTTATTATATTGATCCCAG
	Pr1410	CTGGGATCAATATAATAAAGTACACAGAGAATTTTATCTAAAACGAAATT
**HPr S46D**	Pr1431	GAAGATCTTCTTAAGGAGGCAAATATGGCTTCTAAAGATTTCCA
	Pr1432	CATAACACCCATAATGTCTTTAAGGTTAACTGA
	Pr1433	TCAGTTAACCTTAAAGACATTATGGGTGTTATG
**comE^{D58A} and**	Pr1425	GCTTTATTTCCTAGCTATCGATATTCATGG
**comE^{D58E}**	Pr1426	CCATGAATATCGATAGCTAGGAAATAAAGC
	Pr1429	GAAGATCTTCTTAAGGAGGCAAATATGAAAGTTTTAATTTTAGA
	Pr1430	CCCTCGAGGGTCACTTTTGAGATTTTTTCT
	comE^{D58E}–m3	GCTTTATTTCCTAGAGATCGATATTCATGG
	comE^{D58E}–m2	CCATGAATATCGATCTCTAGGAAATAAAGC
**ΔcomE**	Pr1305	AACATGCTCATCACAAAAGA
	Pr1307	GATTACTGACCGAAAATATTACACAGATGAAATTGTTGGT
	Pr1436	TTTCAGCATTATCCTCTAGATTGACAATTAGCAAGAAATT
	Pr1437	AGCATAAGGAAAGGCTCGAGTCATTCAAATTCCCTCTTAA
**comE-His**	Pr1458	CCGCTCGAGATTTTTGGTCATTAATGGTGATGGTGATGGTGCTTTTGAGATTTTTTCTCTA
	Pr1455	CCGCTCGAGATTTTTGGTCATTATTTATCATCATCATCT
**Primer for generating truncated EMSA probe**		
**P240**	Bio-comCDE F	GATAGAGCATTCGCCTTCTAAG
	Bio-comCDE R	GCTACAAACTGTTCCAATTTAAC
**P179**	Bio-PcomCDE P1	GCAAAGCTGGGAATTTTCCCGGCTTTTTCTTAAAA
	PcomCDE P2-R	CAACTGTCATTTTTTTCTCCCAAA
**P54**	Bio-PcomCDE P3	TAAGTTAGCTTACAAGAAAAAACATTTTAGGAGATTTTATTATGAAAAACACAG
	PcomCDE P3-R	CTGTGTTTTTCATAATAAAATCTCCTAAAATGTTTTTTCTTGTAAGCTAACTTA
**Mut cre1**	Bio-mut cre1 F	ATATAATAGATGCATGTCATGTGAATTCCACGTTGCATCTAATTAAGTACACTTTGGGAGAAAAAAATGACAGTTGAGA
	mut cre1 R	TCTCAACTGTCATTTTTTTCTCCCAAAGTGTACTTAATTAGATGCAACGTGGAATTCACATGACATGCATCTATTATAT
**Mut cre2**	Bio-mut cre2 F	ATATAATAGCAAAGCTGGGAATTTTCCCGGCTTTTTCTTAAAAAAGTACACAACGATAGATATTAGGTAGAATGGAGA
	mut cre2 R	TCTCCATTCTACCTAATATCTATCGTTGTGTACTTTTTTAAGAAAAAAGCCGGGAAAATTCCCAGCTTTGCTATTATAT
**Primer for the expression of recombinant protein**		
**CcpA**	CcpA—F	CGGGATCCATGAATGCCAGATGATACAGTA
	CcpA—R	CCCTCGAGCTATTTACGTTTTCGTGTTGA
**comE and**	comE-F	CGGGATCCATGAAAGTTTTAGAA

**$comE^{D58E}$**	comE-R	CCCTCGAGTCACTTTTGAGATTTTTTCTC
**Primer for qPCR**		
**gyrB**	gyrB-F	GTTCGTATGCGTCCAGGGAT
	gyrB-R	ATACCACGCCCATCATCCAC
**ccpA**	Pr1322	ATGAGTTGGCAGCAGGAGT
	Pr1323	GACGGGTAAAGCGTGAGAT
**comE**	rr12-F	AAGACAACGGGAAAAGTCCG
	rr12-R	AGCTGAGCCACTTCAAATCC
**dprA**	Pr1342	GACATCGCGTCCTTCTTCCA
	Pr1343	TTGCTGGACTTTGTCGTGGT
**comW**	Pr1344	GGCATTGGTTGTCGTAAGGATT
	Pr1345	ACAAGAAATAAACCCCCGATTCA
**comX1**	Pr1346	GGACTGGTAGACGATATTCCACG
	Pr1347	ACGCTTCTGACTTTCCTGCT
**recA**	Pr1348	ATCCAAGCGGACTGAAGCAT
	Pr1349	CCAGGCCATGCGTAAACTTG
**ssbB**	Pr1350	GTTTCTGCTAGTCTGCCCCA
	Pr1351	CTATCGCTGTCAACCGTCGT

Table S3 Differentially expressed genes associated with competence pathways (D39ΔccpA VS D39s)

Regulation status in D39ΔccpA and gene no.[b]	Gene	Description	log2Fold Change[a]	Pvalue[c]
**Early CSP induced genes**				
**SPD_1380**	SPD_1380	hypothetical protein	1.616219	1.01E-05
**SPD_1381**	def2	peptide deformylase	1.78024	2.62E-07
**Late CSP induced genes**				
**SPD_0028**	SPD_0028	phosphoglycerate mutase family protein	-2.07144	5.79E-29
**SPD_0029**	radA	DNA repair protein RadA	-2.28663	1.88E-17
**SPD_0132**	cibB	fratricide two-peptide bacteriocin subunit CibB	-2.53969	8.18E-17
**SPD_0133**	cibA	fratricide two-peptide bacteriocin subunit CibA	-2.55715	0.002211
**SPD_0186**	SPD_0186	hypothetical protein	-2.37821	1.87E-25
**SPD_0683**	SPD_0683	YdbC family protein	-3.07662	7.49E-10
**SPD_0846**	SPD_0846	ABC transporter permease	-2.6082	5.71E-35
**SPD_0981**	SPD_0981	CYTH domain-containing protein	-2.89233	1.57E-05

**SPD_0974**	SPD_0974	gamma-glutamyl-gamma-aminobutyrate hydrolase family protein	-2.47091	0.003449
**SPD_0975**	radC	DNA repair protein RadC	-2.7454	1.55E-07
**SPD_0982**	SPD_0982	GTP pyrophosphokinase family protein	-2.07829	3.26E-16
**SPD_1308**	SPD_1308	aldo/keto reductase	-1.72551	3.99E-11
**SPD_1309**	pgdA	polysaccharide deacetylase family protein	-1.81582	5.17E-27
**SPD_1594**	SPD_1594	XRE family transcriptional regulator	-2.56681	0.001314
**SPD_1595**	SPD_1595	hypothetical protein	-3.10396	6.69E-05
**SPD_1740**	cinA	competence/damage-inducible protein A	-2.02258	5.39E-12
**SPD_1737**	lytA	N-acetylmuramoyl-L-alanine amidase LytA	-1.90387	1.67E-06
**SPD_1738**	dinF	MATE family efflux transporter	-1.77666	9.21E-34
**SPD_1777**	yhaM	3'-5' exoribonuclease YhaM family protein	-2.05201	1.58E-05
**SPD_1778**	rmuC	DNA recombination protein RmuC	-2.14403	0.000417
**SPD_1826**	nadC	carboxylating nicotinate-nucleotide diphosphorylase	-1.70119	7.33E-17
**SPD_1827**	SPD_1827	C4-dicarboxylate ABC transporter	-2.78378	3.11E-05
**SPD_1828**	SPD_1828	ABC transporter ATP-binding protein	-2.32472	3.10E-12
**SPD_1854**	SPD_1854	class I SAM-dependent methyltransferase	-2.04444	4.93E-12
**SPD_0957**	dnaG	DNA primase	-2.16785	6.03E-19
**SPD_0958**	rpoD	RNA polymerase sigma factor RpoD	-2.21022	7.88E-30
**SPD_0959**	SPD_0959	metal-sulfur cluster assembly factor	-2.38501	1.20E-10
**SPD_0983**	ppnK	NAD kinase	-1.66416	1.12E-31
**SPD_0984**	rluD3	RluA family pseudouridine synthase	-1.54058	3.02E-28
**SPD_1121**	SPD_1121	TIGR00341 family protein	-1.65946	5.48E-10
**SPD_1824**	SPD_1824	FtsX-like permease family protein	-2.28103	8.23E-12
**SPD_1858**	comGF	competence protein ComGF	-2.26568	3.06E-07
**SPD_1859**	comGE	hypothetical protein	-2.26632	6.01E-09
**SPD_2028**	cbpD	choline binding-anchored murein hydrolase CbpD	-2.58751	1.43E-13
**SPD_0843**	comEA	ComEA family DNA-binding protein	-2.24234	0.001551
**SPD_0844**	comEC	DNA internalization-related competence protein ComEC/Rec2	-2.32452	4.05E-31

**SPD_0865**	coiA	competence protein CoiA	-1.83684	2.14E-05
**SPD_1711**	ssbB	single-stranded DNA-binding protein	-2.77504	4.83E-09
**SPD_1739**	recA	recombinase RecA	-1.8066	7.43E-25
**SPD_1857**	comGG	hypothetical protein	-2.30235	9.88E-12
**SPD_1860**	comGD	type II secretion system GspH family protein	-2.29934	4.95E-10
**SPD_1861**	comGC	comG operon protein ComGC	-2.34829	8.51E-13
**SPD_1862**	comGB	type II secretion system F family protein	-2.31331	4.87E-06
**SPD_1863**	comGA	GspE/PulE family protein	-2.74165	1.49E-43
**SPD_2034**	comFC	ComF family protein	-1.69451	4.48E-14
**SPD_2035**	comFA	DEAD/DEAH box helicase	-1.74127	6.00E-08
**SPD_1122**	dprA	DNA-processing protein DprA	-2.55795	2.42E-08
**Delayed CSP induced genes**				
**SPD_1526**	SPD_1526	membrane protein	-1.5124	1.46E-08
**SPD_0458**	hrcA	heat-inducible transcriptional repressor HrcA	1.555345	3.58E-05
**SPD_0459**	grpE	nucleotide exchange factor GrpE	1.987324	1.18E-07
**SPD_0460**	dnaK	molecular chaperone DnaK	1.808327	8.18E-13
**SPD_0461**	dnaJ	molecular chaperone DnaJ	1.533612	2.83E-21
**SPD_1710**	groES	co-chaperone GroES	-1.54957	2.98E-05

[a]Log2 fold change in gene expression as assessed by RNA-seq.

[b]The reference genome under GenBank accession numbers NC_008533.2 and CP027540.1.

[c]Adjusted P values, P values were adjusted using the Benjamini and Hochberg method.

Table S4 Differentially expressed genes associated with PTS transporter (D39ΔccpA VS D39s)

**Regulation status in D39ΔccpA and gene no.**[b]	**Gene**	**Description**	**log2Fold Change**[a]	**Pvalue**[c]
**SPD_0561**		PTS galactitol transporter subunit IIC	4.257115	4E-167
**SPD_0502**	bglF	PTS glucose transporter subunit IIA	1.8221	4.05E-08
**SPD_0661**	malT	PTS glucose transporter subunit IIABC	5.450241	1.08E-82
**SPD_0264**	manL	PTS mannose transporter subunit IIAB	2.079716	3.82E-18
**SPD_0262**	manN	PTS mannose/fructose/sorbose transporter family subunit IID	1.824718	6.72E-35

**SPD_0068**	gadE	PTS mannose/fructose/sorbose transporter family subunit IID	5.269174	4.7E-185
**SPD_1989**		PTS mannose/fructose/sorbose transporter family subunit IID	1.942866	0.000168
**SPD_0297**		PTS mannose/fructose/sorbose transporter family subunit IID	2.513531	3.67E-06
**SPD_0263**	manM	PTS mannose/fructose/sorbose transporter subunit IIC	2.036482	1.28E-16
**SPD_0067**	gadW	PTS mannose/fructose/sorbose/N-acetylgalactosamine transporter subunit IIC	5.099736	2.45E-92
**SPD_1990**		PTS mannose/fructose/sorbose/N-acetylgalactosamine transporter subunit IIC	1.981544	0.000192
**SPD_0296**		PTS mannose/fructose/sorbose/N-acetylgalactosamine transporter subunit IIC	3.006299	2.42E-08
**SPD_0069**	gadF	PTS sugar transporter subunit IIA	5.064813	4.1E-104
**SPD_0559**		PTS sugar transporter subunit IIA	4.139283	0.028442
**SPD_0560**		PTS sugar transporter subunit IIB	3.209695	0.000131
**SPD_0066**	gadV	PTS system mannose/fructose/N-acetylgalactosamine-transporter subunit IIB	5.277276	2.3E-137
**SPD_0295**		PTS system mannose/fructose/N-acetylgalactosamine-transporter subunit IIB	2.389446	0.031061
**SPD_1496**	nanP	PTS transporter subunit EIIC	4.606194	1.82E-86

[a]Log2 fold change in gene expression as assessed by RNA-seq.

[b]The reference genome under GenBank accession numbers NC_008533.2 and CP027540.1.

[c]Adjusted P values, P values were adjusted using the Benjamini and Hochberg method.

References

1. Zhang J, Ye W, Wu K, Xiao S, Zheng Y, Shu Z, Yin Y, Zhang X: **Inactivation of Transcriptional Regulator FabT Influences Colony Phase Variation of Streptococcus pneumoniae**. *mBio* 2021, **12**(4):e0130421.

2. Biswas I, Jha JK, Fromm N: **Shuttle expression plasmids for genetic studies in Streptococcus**

mutans. *Microbiology (Reading)* 2008, **154**(Pt 8):2275-2282.

3. Prudhomme M, Attaiech L, Sanchez G, Martin B, Claverys JP: **Antibiotic stress induces genetic transformability in the human pathogen Streptococcus pneumoniae**. *Science* 2006, **313**(5783):89-92.

4. Pestova EV, Morrison DA: **Isolation and characterization of three Streptococcus pneumoniae transformation-specific loci by use of a lacZ reporter insertion vector**. *J Bacteriol* 1998, **180**(10):2701-2710.

5. Yother J, McDaniel LS, Briles DE: **Transformation of encapsulated Streptococcus pneumoniae**. *J Bacteriol* 1986, **168**(3):1463-1465.

6. Eberhardt A, Wu LJ, Errington J, Vollmer W, Veening JW: **Cellular localization of choline-utilization proteins in Streptococcus pneumoniae using novel fluorescent reporter systems**. *Mol Microbiol* 2009, **74**(2):395-408.
